# CD103^+^CD8^+^ tissue‐resident memory T lymphocytes of melanoma boost anti‐tumour immunity and predict immunotherapy outcomes

**DOI:** 10.1002/ctm2.70464

**Published:** 2025-09-23

**Authors:** Tianyi Zhang, Junquan Song, Yinlam Li, Kangjie Shen, Jiangying Xuan, Yuan Gao, Lili Lu, Zhi Pang, Lu Wang, Yang Yang, Zixu Gao, Qianrong Hu, Yu Zhu, Chenlu Wei, Shaoluan Zheng, Rongkui Luo, Yingyong Hou, Yuhong Zhou, Chuanyuan Wei, Jianying Gu

**Affiliations:** ^1^ Department of Plastic Surgery Zhongshan Hospital, Fudan University Shanghai PR China; ^2^ Clinical Center for Biotherapy Zhongshan Hospital, Fudan University Shanghai PR China; ^3^ Department of Plastic and Reconstructive Surgery Zhongshan Hospital (Xiamen), Fudan University Shanghai PR China; ^4^ Department of Pathology Zhongshan Hospital, Fudan University Shanghai PR China; ^5^ Department of Medical Oncology Zhongshan Hospital, Fudan University Shanghai PR China

**Keywords:** immunotherapy, melanoma, TIL, tissue‐resident memory T lymphocytes

## Abstract

**Background:**

Immunotherapy has revolutionised melanoma treatment, providing significant clinical benefits by reactivating the anti‐tumour immune system. CD8^+^ tissue‐resident memory T lymphocytes (CD8^+^ TRM) have emerged as crucial mediators of anti‐tumour immunity, while their specific role in melanoma remains poorly understood.

**Methods:**

Following CD8^+^CD45.1^+^ OT‐1 cell adoptive transfer into CD45.2^+^ mice, we employed magnetic separation to purify and analyse resident memory CD8^+^ T cells (TRM). We use multiple immunohistochemistry (mIHC) to evaluate the spatial distribution of CD8^+^ TRM in ZS melanoma cohort. Additionally, the biological function of CD8^+^ TRM and their impact on anti‐tumour immunity are explored using scRNA sequencing and spatial transcriptomics, coupled with in vivo/in vitro experiments. Finally, CD8^+^ TRM utility as an immunotherapy response predictor is examined across several independent cohorts.

**Results:**

CD8^+^ TRM demonstrates potent tumour‐killing capabilities in melanoma, with CD103 as a distinctive marker. High CD103^+^CD8^+^ TRM infiltration in tumour tissues strongly correlates with improved prognosis in melanoma patients. In vivo adoptive transfer of CD103^+^CD8^+^ TRM effectively inhibits melanoma progression. Mechanistically, CD103 activates the integrin‐dependent PI3K/AKT signalling cascade, promoting both proliferation and anti‐tumour effector functions of CD8^+^ TRM. Notably, CD103^+^CD8^+^ TRM preferentially localises within tertiary lymphoid structures (TLS), and its adoptive transfer promotes TLS formation. Clinically, CD103^+^CD8^+^ TRM is enriched in immunotherapy‐responsive patients and serves as a strong predictor for immune checkpoint blockade (ICB) treatment outcomes.

**Conclusions:**

CD103^+^ CD8^+^ TRM cells in melanoma play a key role in the anti‐tumour immune process and can also be used as a reliable predictor of immunotherapy efficacy.

**Key points:**

- CD103 is a reliable marker of tissue‐resident memory (TRM) CD8^+^ T cells in melanoma.
- CD103^+^CD8^+^ TRM cells exhibit potent anti‐tumour immune activity.
- CD103^+^CD8^+^ TRM cells predict favourable responses to immunotherapy in melanoma.

## Graphical abstract

1. CD103 is a reliable marker of tissue‐resident memory (TRM) CD8^+^ T cells in melanoma.
2. CD103^+^CD8^+^ TRM cells exhibit potent anti‐tumour immune activity.
3. CD103^+^CD8^+^ TRM cells predict favourable responses to immunotherapy in melanoma.

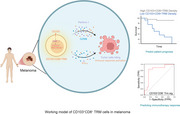

## 1 INTRODUCTION

Immune checkpoint blockades (ICBs) have been widely demonstrated to improve prognosis and extend survival across various cancers,^1^, ^2^ highlighting the efficacy of reactivating anti‐tumour immunity as a therapeutic strategy. T lymphocytes, particularly CD8^+^ T cell who plays a critical role in immune surveillance and destruction of mutated tumour cells, as evidenced by numerous studies.^3^, ^4^ As research into T lymphocytes has advanced, many markers associated with their functional states have been identified, including exhaustion markers (e.g., PD‐1 and CTLA‐4), effector markers (e.g., GZMB and perforin) and stemness markers such as TCF1.^5^ A rapid development of single‐cell sequencing and mass cytometry, alongside advancements in analytical techniques, has deepened our understanding of T‐cell subsets, revealing the distinct states, functions and markers of CD8^+^ T cells.^6^, ^7^ The success of ICB therapies has also demonstrated that even T cells expressing exhaustion markers like PD‐1 retain potent tumour‐killing potential,^8^ which can be effectively reactivated by ICB to inhibit tumour progression. Therefore, to deeply understand the role and function of immune cell subsets in the complex and changeable immune microenvironment, it is necessary to carry out comprehensive and detailed research on them.

In the local immune microenvironment of tumours, diverse immune cell types play indispensable roles during immune responses,^9^ coordinating to create a harmonious interplay. Lymphocytes are critical players in both adaptive immune responses^10^ and their crosstalk with innate immunity,^11^ and they are a focal point of interest in tumour immunology.^12^ Lymphocyte differentiation, maturation, activation and deactivation represent a complex, orderly regulatory process. Traditionally, lymphocytes involved in immune responses were thought to be recruited from peripheral lymphoid organs into non‐lymphoid tissues via the bloodstream. However, advancements in phenotyping techniques have brought increasing attention to tissue‐resident lymphocytes within the local microenvironment.^13^, ^14^, ^15^

Studies suggest that memory lymphocytes with tissue‐resident phenotypes are key players in immune surveillance and cytotoxicity, characterised by the upregulation of residency‐related functional markers.^16^, ^17^ Furthermore, the maintenance of immune memory and surveillance functions within the local microenvironment is primarily attributed to tissue‐resident rather than circulating memory lymphocytes, challenging the traditional ‘migration'paradigm.^18^ CD8^+^ tissue‐resident memory (TRM) subset are widely distributed across various tissues, including the skin, liver, intestinal mucosa and tumour tissues.^19^ The CD8^+^ TRM plays a critical role in chronic infections and tumour immune surveillance. However, the distribution of CD8^+^ TRM within melanoma tissues, their prognostic value, the specific marker of CD8^+^ TRM and the mechanisms by which they mediate tumour surveillance and regulate the local microenvironment remain poorly understood.

In this study, we used adoptive transfer models to identify and isolate CD8^+^ TRM in melanoma. Through a combination of advanced methodologies, we investigated their phenotypes and critical roles within the immune microenvironment and found that the CD103 molecule is the most critical marker of the CD8^+^ TRM. Our findings demonstrate the potent tumour‐killing capability of CD8^+^ TRM and their significant predictive value for immunotherapy response.

## 2 MATERIALS AND METHODS

### 2.1 Patient selection and follow‐up

One hundred and twenty‐three melanoma patients who underwent complete tumour resection at the Plastic Surgery Department of Zhongshan Hospital Affiliated to Fudan University were randomly divided into a discovery cohort and a verification cohort. The specific criteria for inclusion of patients are as follows: first, they have been diagnosed with melanoma by histology and pathology; second, they have not received radiotherapy, chemotherapy or immunotherapy before surgery; third, they have no history of other malignant tumours or have been diagnosed at the same time; fourth, they have achieved complete resection and microscopic confirmation of negative cutting margin; fifth, they have complete clinical pathology and follow‐up data.

The discovery cohort consisted of 49 patients, and the validation cohort included 74 patients. Detailed clinical baseline characteristics are presented in Table 1.

**TABLE 1:** Clinical characteristics of melanoma patients at Zhongshan Hospital.

	Discovery set (*n* = 49)					Validation set (*n* = 74)				
	Patients		CD8^+^CD103^+^T cells			Patients		CD8^+^CD103^+^T cells		
	NO	%	Low	High	*p*	NO	%	Low	High	*p*
**All patients**	49	100.0	25	24		74	100.0	37	37	
**Age**					1.000					.346
<60	18	36.7	9	9		31	41.9	18	13	
≥3.	31	63.3	16	15		43	58.1	19	24	
**Gender**					1.000					.161
Female	22	44.9	11	11		33	44.6	20	13	
Male	27	55.1	14	13		41	55.4	17	24	
**Anatomic site**					.261					.308
Other	14	28.6	9	5		13	17.6	6	7	
Acral	19	38.8	7	12		39	52.7	17	22	
Trunk	16	32.7	9	7		22	29.7	14	8	
**Histologic type**					.280					.179
Superficial spreading	14	28.6	7	7		17	23.0	11	6	
Nodular	12	24.5	7	5		13	17.6	6	7	
Acral	13	26.5	4	9		26	35.1	9	17	
Lentigo maligna	10	20.4	7	3		18	24.3	11	7	
**Breslow depth (mm)**					.322					.100
≤1	29	59.2	17	12		32	43.2	12	20	
>2	20	40.8	8	12		42	56.8	25	17	
**Clark level**					.874					.162
I—III	22	44.9	12	10		35	47.3	14	21	
IV–V	27	55.1	13	14		39	52.7	23	16	
**Ulceration**					.625					.155
Absent	43	87.8	23	20		65	87.8	35	30	
Present	6	12.2	2	4		9	12.2	2	7	
**Lymph nodes metastasis**					.632					.344
No	45	91.8	22	23		62	83.8	29	33	
Yes	4	8.2	3	1		12	16.2	8	4	
**Distant metastasis**					.273					1.000
No	41	83.7	19	22		55	74.3	27	28	
Yes	8	16.3	6	2		19	25.7	10	9	
**Clinical stage**					.196					.346
I–II	38	77.6	17	21		43	58.1	19	24	
III–IV	11	22.4	8	3		31	41.9	18	13	

Statistical methods: chi‐square test (with Fisher's exact test employed in certain cases).

### 2.2 Ethical approval

This study was approved by the Biomedical Research Ethics Committee of Zhongshan Hospital (Approval No. 2023‐036) and the Institutional Animal Care and Use Committee (IACUC) of Zhongshan Hospital (Approval No. 2023‐293).

### 2.3 Univariate and multivariate prognostic analyses related to OS and DFS

We incorporated confounding factors such as age, gender, metastasis, stage, histological subtype, as well as the density of the TRM subpopulation, and conducted both univariate analysis and multivariate Cox regression analysis. Based on the characteristics of the samples, the appropriate statistical method was selected for univariate analysis. For dichotomous variables, the two‐sample *t*‐test was employed if the samples met criteria for normal distribution; otherwise, the Mann–Whitney *U*‐test was performed. For polytomous variables, one‐way analysis of variance (ANOVA) was applied if the samples met criteria for normal distribution; otherwise, the Kruskal–Wallis test was utilised. Shapiro–Wilk test was employed to assess whether samples met the criteria for normal distribution.

### 2.4 Tissue microarray construction

Tissue microarray (TMA) construction followed protocols described in our previous studies.^20^ Briefly, the specimens removed by surgery must be fixed in formalin, then embedded in paraffin (FFPE), and then stained with haematoxylin–eosin (H&E) to select representative tumour areas. Duplicate 1‐mm diameter cores were taken from each tumour sample to construct the TMA, which was subsequently used for multiplex immunofluorescence staining.

### 2.5 mIHC and image analysis

Using standard primary antibodies and TSA 7 colour kits, step‐by‐step multiple immunohistochemistry (mIHC) operations were performed on FFPE tissue sections with a thickness of 4 µm (YM0086Plus‐100T, Ningbo Yangming Medical Laboratory), following the manufacturer's protocol. After staining, nuclei were labelled with 4′,6‐Diamidino‐2‐Phenylindole (DAPI).

Sections were deparaffinised, subjected to antigen retrieval, and blocked. Primary anti‐CD8 antibodies (Cat#ab245118, Cat#ab217344, Abcam) were incubated at room temperature for 1.5 h, followed by incubation with an horseradish peroxidase (HRP)‐conjugated universal secondary antibody (YM054, Ningbo Yangming Medical Laboratory) for 50 min. Labelling was performed using TYR‐520Plus fluorescent dye according to the instructions given by the manufacturer for 10 min. Subsequently, the slides were washed with tris buffered saline tween (Buffer) (TBST) buffer and additional antigen retrieval operations were performed using ethylenediaminetetraacetic acid (EDTA) (pH 9.0). The specific operation method is to place the container containing EDTA into a microwave oven and heat it at 20% of the maximum power for 15 min.

Subsequent rounds of staining were performed for CD103 (Cat#ab224202, Abcam) and TYR‐570Plus, and CD19 (Cat#ab245235, Cat#ab134114, Abcam) and TYR‐690Plus, with phosphate‐buffered saline (PBS) washes between steps. After completing all staining steps, slides were counterstained with DAPI and mounted with anti‐fade mounting medium. Imaging was performed using the Aperio Versa 8 Tissue Imager (Leica), and images were analysed using Indica HALO software (version 3.3.2541.301).

### 2.6 Isolation, magnetic sorting and activation of tumour‐infiltrating lymphocytes

Tumour tissue is cut into pieces and then digested with type IV collagenase (Cat#C5138, Sigma), DNase I (Cat#EN0521, Thermo Scientific) and hyaluronidase (Cat#H3506, Sigma), followed by density gradient centrifugation (Figure S1B). The specific magnetic sorting strategy is described in detail in the main text. Briefly, different subsets were magnetically labelled based on their surface markers (Cat#130‐121‐223; Cat#130‐124‐089; Cat#130‐111‐684; Cat#130‐090‐485, Miltenyi) for sorting (Cat#130‐042‐401, Cat#130‐042‐301, Miltenyi).

**FIGURE 1:**
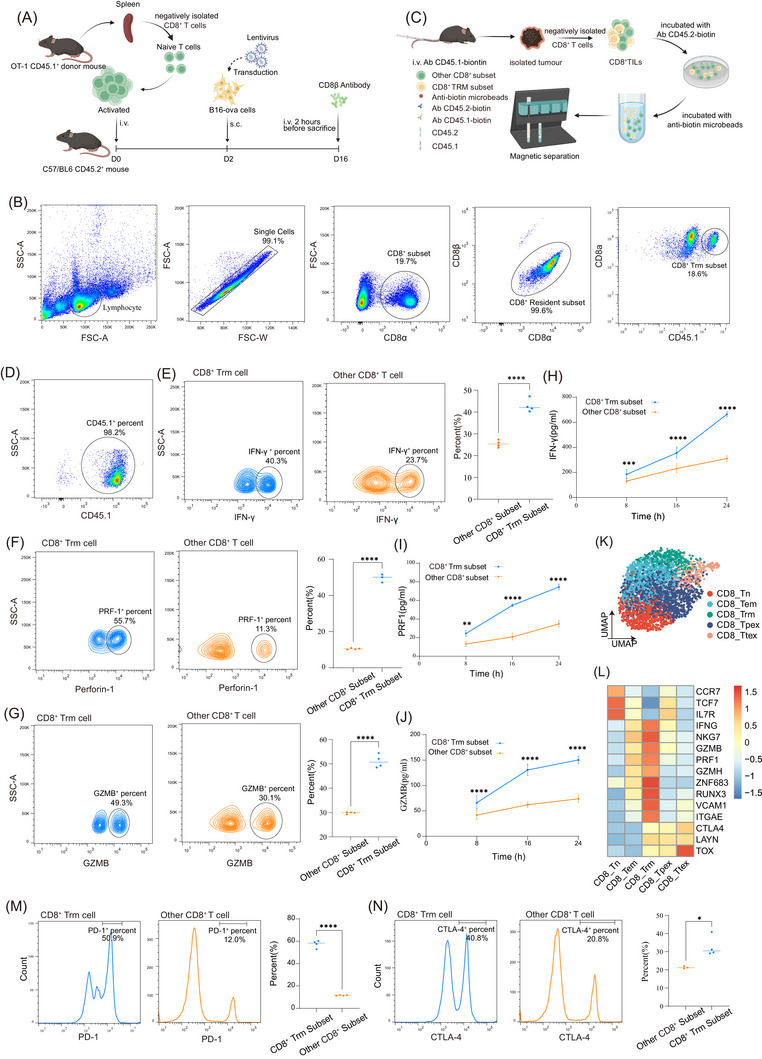
CD8^+^ tissue‐resident memory (TRM) exhibits potent tumour‐killing role. (A) Animal model construction: Lymphocytes were obtained from the spleen of CD45.1^+^ OT‐1 mice, cultured and expanded in vitro, and then intravenously injected into CD45.2^+^ C57/BL6 recipient mice, with 5 × 10^4^ cells injected per mouse. B16‐OVA cells were subsequently subcutaneously injected to establish a tumour‐bearing model. Two hours before euthanasia, a 1:100 dilution of anti‐CD8β fluorescent antibody was injected intravenously into the mice. (B) Flow cytometry gating strategy for TRM subset identification. (C) Magnetic sorting strategy for CD8^+^ TRM and other CD8^+^ cells: Two hours before euthanasia, a 1:100 dilution of anti‐CD45.1‐biotin antibody was injected intravenously into tumour‐bearing mice. After tumour tissue was harvested, CD8^+^ T lymphocytes were isolated using a negative selection strategy (Figure S1B). The isolated CD8^+^ T lymphocytes were incubated with anti‐CD45.2‐biotin antibody in vitro, followed by incubation with magnetic beads. Magnetic sorting was then performed. Positive selection identified other CD8^+^ cells, while negative selection identified CD8^+^ TRM cells. (D) Efficiency of magnetic sorting strategy. (E) Flow cytometry detection of IFN‐γ expression. (F) Flow cytometry detection of perforin‐1 expression. (G) Flow cytometry detection of GZMB expression. (H) In vitro ELISA experiment for IFN‐γ secretion in CD8^+^ TRM and other CD8^+^ cells post sorting, stimulation and culture. (I) In vitro ELISA experiment for Perforin‐1 secretion in CD8^+^ TRM and other CD8^+^ cells post sorting, stimulation and culture. (J) In vitro ELISA experiment for GZMB secretion in CD8^+^ TRM and other CD8^+^ cells post sorting, stimulation and culture. (K) Single‐cell sequencing analysis: UMAP plot of CD8^+^ T cells (*n* = 2890). (L) Heatmap of marker genes for different CD8 subsets: naïve CD8^+^ T cells (CD8_Tn), effector memory CD8^+^ T cells (CD8_Tem), tissue‐resident memory CD8^+^ T cells (CD8_Trm), progenitor exhausted CD8^+^T cells (CD8_Tpex) and exhausted CD8^+^ T cells (CD8_Tex). (M, N) Expression of surface immune checkpoints (PD‐1, CTLA‐4) on CD8^+^ TRM and other CD8^+^ cells.

**FIGURE 2:**
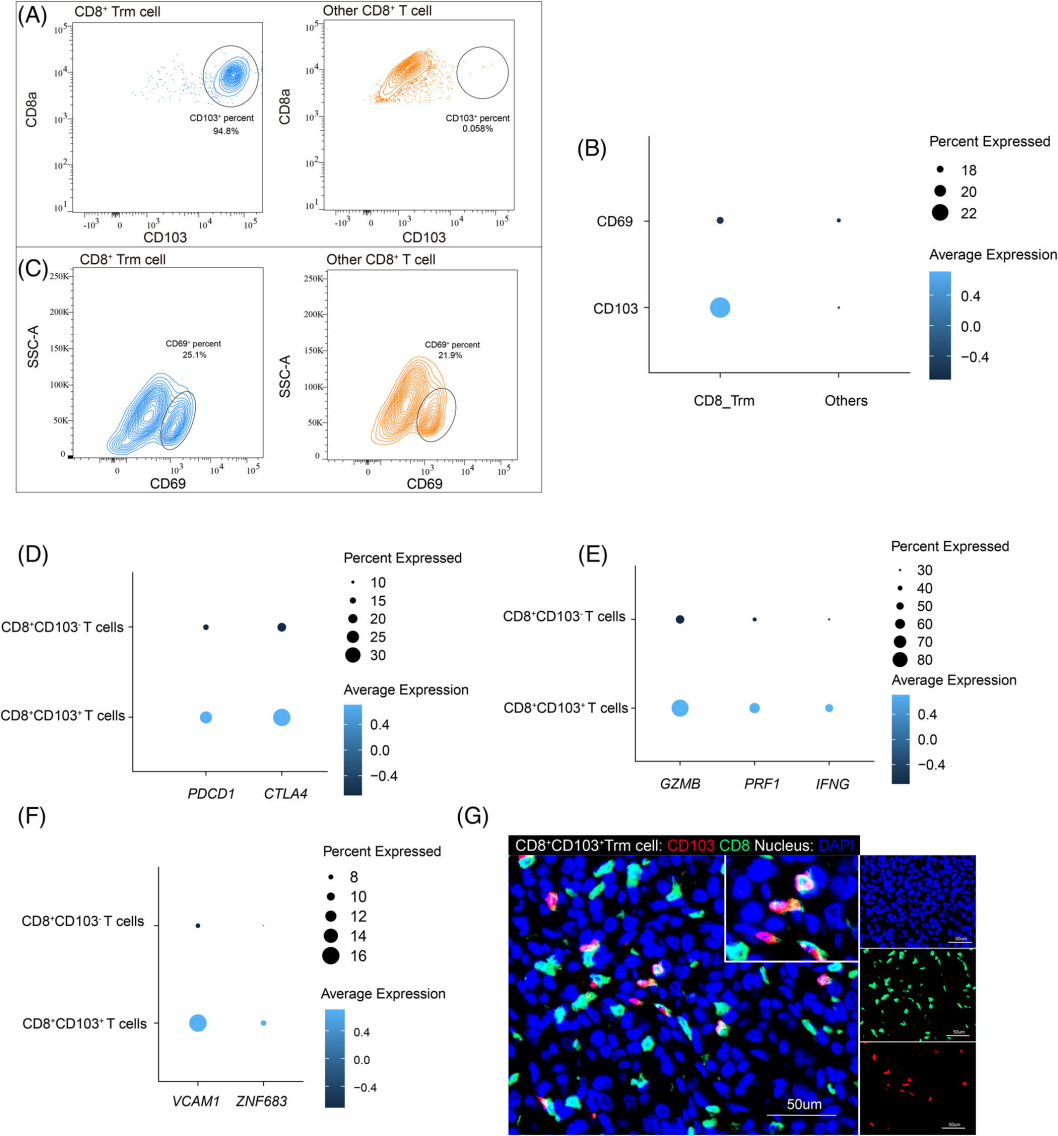
CD103 is the specific marker for CD8^+^ tissue‐resident memory (TRM) in melanoma. (A) Surface expression of CD103 (ITGAE) on TRM and others. (B) Single‐cell sequencing analysis of CD103 (ITGAE) and CD69 gene expression in TRM and others. (C) Surface expression of CD69 on TRM and others. (D) Single‐cell sequencing analysis of expression of ‘exhaustion’‐related markers in CD8^+^CD103^+^ cells and others. (E) Single‐cell sequencing analysis of expression of effector‐related markers in CD103^+^CD8^+^ cells and others. (F) Single‐cell sequencing analysis of the expression of markers of tissue‐resident memory subsets in CD103^+^CD8^+^ cells and others. (G) Typical immunofluorescence picture of the CD103^+^CD8^+^ cells.

For T lymphocytes requiring reactivation, cells were activated using magnetic microbeads (Cat#11452D, Gibco) and IL‐2 (Cat#I0523, Sigma). When necessary, magnetic microbeads were separated using a magnetic rack (Cat#12303D, Invitrogen). The T cells used in each part will be reflected in the schematic diagrams of the respective figures. The source of T cells in Figure 2 is the same as that in Figure 1. The T cells used in Figures 1 and 2 were directly detected for phenotype without expansion and reactivation. In contrast, the T cells used in other figures for in vitro or in vivo experiments were subjected to brief activation and expansion before being used for co‐culture experiments or adoptive transfer experiments in vivo. When AKT inhibitors are required to treat T cells, the treatment conditions and concentrations are as follows. The MK2206 inhibitor (4.8 mg) is dissolved in 1 mL of dimethyl sulfoxide (DMSO) to prepare a 10 mM solution, which is then diluted to 2 µM with culture medium for use. The control group's DMSO solution is diluted with culture medium in the same proportion.

### 2.7 Flow cytometry

Cell suspensions requiring red blood cell lysis were treated with red blood cell (RBC) lysis buffer (Cat#00‐4333‐57, Invitrogen). Single‐cell suspensions were washed and then resuspended in PBS buffer containing .1% bovine serum albumin (BSA). Surface markers were then stained with fluorescence‐conjugated antibodies at 4°C for about 40 min. Intracellular proteins were stained using fluorescence‐conjugated antibodies and a fixation/permeabilisation kit (Cat#562574, BD Biosciences), following stimulation with blocking agents (Cat#550583, BD Biosciences) or reactivation with CD3/CD28 magnetic microbeads (Cat#11452D, Gibco) as needed. Fluorescence‐activated cell sorting (FACS) data were collected using a BD FACS Celesta flow cytometer and analysed using FlowJo V10.0 software.

The antibodies used in this section of the experiment include: BV510 Rat Anti‐Mouse CD8b (Cat#740155, BD); PE Rat Anti‐Mouse CD8b (Cat#550798, BD); FITC Rat Anti‐Mouse CD8a (Cat#561966, BD); PerCP‐Cy5.5 Mouse Anti‐Mouse CD45.2 (Cat#552950, BD); APC‐Cy7 Mouse Anti‐Mouse CD45.1 (Cat#560579, BD); APC Mouse Anti‐Mouse CD45.1 (Cat#558701, BD); PE Hamster Anti‐Mouse CD103 (Cat#566844, BD); APC Anti‐Mouse CD279 (PD‐1) Antibody (Cat#135251, Biolegend); APC Anti‐Mouse CD152 (CTLA4) Antibody (Cat#106309, Biolegend); APC Anti‐Human/Mouse Granzyme B Recombinant Antibody (Cat#372203, Biolegend); APC Anti‐Mouse IFN‐γ Antibody (Cat#505809, Biolegend); PE Anti‐Mouse IFN‐γ Antibody (Cat#505807, Biolegend); APC Anti‐Mouse Perforin Antibody (Cat#154303, Biolegend); APC Anti‐Mouse CXCL13 Antibody (Cat#17‐7981‐82, Thermo).

### 2.8 Survival analysis and immune infiltration analysis in the TCGA SKCM cohort

We downloaded sequencing data of samples in the cancer genome atlas (TCGA) skin cutaneous melanoma (SKCM) cohort from the official UCSC database, comprising a total of 472 sequencing datasets, which included one normal tissue sequencing dataset and 471 melanoma sample sequencing datasets. The survival data and clinical information of this cohort were also obtained from the UCSC official database. After comparison, 457 melanoma sample sequencing datasets were matched with corresponding survival information. Subsequently, the samples were scored according to the expression level of CD103^+^CD8^+^ TRM signature gene set. The samples were divided into two groups according to whether their scores were above the median of the total score. Survival analysis and immune infiltration analysis were then performed. The immune infiltration analysis was conducted using the CIBERSORT script for local analysis. Spearman correlation analysis was used to study the correlation between ITGAE gene expression levels and scores of 22 immune cell types. Figures are generated using the ggplot2 R package (version 3.5.1.9000). Survival analysis is completed through the survival R package (version 3.8‐3) and survminer R package (version 0.4.9) of the R, respectively.

### 2.9 Cell line construction and transfection

The B16F10 cell line was purchased from Cell Bank of China Academy of Sciences (Shanghai) and cultured according to recommended conditions. The lentivirus vector used in this study was provided by Shanghai Obi Pharmaceutical Technology Co., Ltd. Successful expression of luciferase or ova in the B16‐Luci or B16‐OVA cell line was confirmed by observing green fluorescent protein (GFP) fluorescence under a microscope.

### 2.10 Animal models and treatment

Female CD45.2^+^ C57BL/6 mice (5–6 weeks old) were purchased from Beijing Vital River Laboratory Animal Technology, while female CD45.1+ OT‐1 C57BL/6 mice were obtained from Cyagen Biosciences. All mice were raised at the Experimental Animal Center of Zhongshan Hospital Affiliated to Fudan University, which is a specific pathogen barrier (SPF) facility.

In the adoptive cell animal model characterising the TRM subpopulation, B16F10‐OVA cells (1 × 10⁶) were subcutaneously injected into the right flank of CD45.2^+^ C57BL/6 mice. A total of 5 × 10^4^ CD45.1^+^ OT‐1 lymphocytes were adoptively transferred via tail vein injection into CD45.2^+^ C57BL/6 mice. When performing in vivo experiments with the adoptive transfer of two different types of T lymphocytes for treatment, the subcutaneous tumour model was established by subcutaneous injection of 5 × 10^5^ B16Luci cells, and the intratumoural cell transfer dose was set at an effector‐to‐target ratio of 1:1 for the transfer of the two types of T cells. For the lung metastasis model, B16‐Luci cells were transferred via tail vein injection, with the same cell transfer dose as that used in the subcutaneous tumour model. When cell transfer was combined with anti‐PD‐1 treatment, the cell transfer dose remained the same as before, and the transfer method was intratumoural injection. The anti‐PD‐1 drug (clone number: RMP1‐14) was administered at a dose of 10 mg/kg via intraperitoneal injection. Bioluminescent imaging was performed using the IVIS SpectrumCT system, and images were standardised using Living Image software (Perkin Elmer).

### 2.11 Functional enrichment analysis

Sequencing data (GSE194383) were obtained from GEO database. Differentially expressed genes (DEGs) of different cell groups were analysed using Limma–Voom algorithm of Limma package (3.60.3), with the following criteria: |log2(Fold Change)| ge)| imma *p* < .05. Gene ontology (GO) and kyoto encyclopedia of genes and genomes (KEGG) enrichment analyses were performed using the clusterProfiler (4.12.0) package. Results were then showed using the enrichplot (1.24.0) package. Gene set enrichment analysis (GSEA) was conducted using the GSEA (1.2) package, with *q* < .25, NES > 1 and *p* < .05 considered statistically significant.

### 2.12 Western blot

Proteins in cells or tissues were extracted with radio‐immunoprecipitation assay (Buffer) (RIPA) lysis buffer added with protease and phosphatase inhibitors. The extracted lysate was centrifuged at a speed of 12 000 × *g* for 15 min at 4°C. Then the supernatant was collected for subsequent analysis, and the protein concentration was determined using the bicinchoninic acid assay (BCA) protein assay kit.

An equal amount of protein, generally 20 µg, was taken from each sample, separated by sodium dodecyl sulphate‐polyacrylamide gel electrophoresis (SDS‐PAGE) gel electrophoresis and transferred to a polyvinylidene fluoride (PVDF) membrane. After blocking with 5% non‐fat milk at room temperature for 1 h, membranes were incubated overnight at 4°C with primary antibodies (diluted 1:1000). Following TBST washes, membranes were treated with HRP‐linked secondary antibodies (1:5000) for 1 h. Finally, enhanced chemiluminescence, or ECL reagent, is used for colour development, and the bands are observed through a gel imaging system. The antibodies used in this section of the experiment include: Akt (pan) (C67E7) Rabbit mAb (Cat#4691, CST); Phospho‐Akt (Ser473) (D9E) XP® Rabbit mAb (Cat#4060, CST); Glyceraldehyde‐3‐phosphate dehydrogenase (GAPDH) Rabbit mAb (Cat#ab181602, Abcam); Goat Anti‐Rabbit IgG H&L (HRP; Cat#ab205718, Abcam).

### 2.13 Analysis of single‐cell sequencing data

ScRNA sequencing data for human melanoma were acquired from our previously published study. The raw gene expression matrix was processed and integrated using R package Seurat (v4.3.0). We eliminated cells that expressed fewer than 200 genes or more than 6000 genes. Cells with mitochondrial gene counts exceeding 15% of the total gene number were also excluded. The Harmony (v1.0) algorithm was used to integrate sequencing data from different datasets.

Normalised and scaled data were subjected to principal component analysis (PCA) using the top 2000 highly variable genes. We used the first 15 principal components to embed UMAP. In order to clarify the functional characteristics of each cluster, I used the FindAllMarkers function to identify DEGs. The UCell (v1.1.0) package was used to calculate the CD8^+^CD103^+^ TRM signature score, defined by markers including *CD8A*, *ITGAE*, *RUNX3*, *GZMK*, *CXCL13* and *ZNF683*. We performed the receiver operating characteristic (ROC) curve analysis using the R package pROC (version 1.18.0).^21^

### 2.14 Cell–cell communication analysis

To explore potential interactions between CD103^+^CD8^+^ T cells and other cells, we used the ‘CellChat’ R package.^22^ Single‐cell transcriptomic data were normalised, and cell types were annotated using Seurat (v4.3.0). We constructed a CellChat object and inferred ligand–receptor interactions based on the built‐in human interaction database. Communication probabilities were calculated, and interaction networks were visualised.

### 2.15 Spatial transcriptomics analysis

Methods for spatial transcriptomic sequencing and analysis of human melanoma samples were described in our previous publication. Briefly, after quality control, the data were then normalised through the SCTransform function of the R package Seurat to identify highly variable genes. Immune cell densities were calculated using the MCPcounter (v1.2.0) tool.

CD8^+^CD103^+^ TRM and tertiary lymphoid structures (TLS) signature scores were computed using the UCell (v1.1.0) package. The CD103^+^CD8^+^ TRM signature included *CD8A*, I*TGAE*, *RUNX3*, *GZMK*, *CXCL13* and *ZNF683*, while the TLS signature included *CCL2*, *CCL3*, *CCL4*, *CCL5*, *CCL8*, *CCL18*, *CCL19*, *CCL21*, *CXCL9*, *CXCL10*, *CXCL11*, *CXCL13*, *IRF5* and *KYNU*.

### 2.16 Statistical analysis

Group differences were assessed using one‐way ANOVA for normally distributed data. For two‐group comparisons, either unpaired *t*‐tests or Mann–Whitney *U*‐tests were applied based on data distribution. Categorical variables were compared via chi‐square tests. Survival analyses employed Kaplan–Meier estimates and log‐rank tests. All analyses were two‐tailed with a significance threshold of *p* < .05. Results are shown as mean ± SD. Statistical tests were conducted using GraphPad Prism (v9.0.1) and R (v4.4.0).

## 3 RESULTS

### 3.1 CD8^+^ TRM exhibits potent tumour‐killing role

To characterise the genuine CD8^+^ TRM subset in the melanoma microenvironment, we utilised an adoptive cell transfer model, following a methodology similar to that employed by previous researchers.^18^, ^19^, ^23^ First, we constructed a B16F10‐OVA melanoma cell line overexpressing the ovalbumin (OVA) antigen using lentiviral transduction (Figure S1A). Genetically engineered CD8^+^ T cells were obtained from the spleens of CD45.1^+^ OT‐1 mice using a combination of gradient centrifugation and magnetic sorting (Figure S1B), and the efficiency of this sorting strategy was validated (Figure S1C). Next, CD8^+^CD45.1^+^ OT‐1 cells were intravenously transferred into CD45.2^+^ recipient mice, followed by subcutaneous implantation of B16‐OVA cells. Two hours prior to euthanasia, mice were intravenously injected with fluorescently labelled anti‐CD8β antibody to label circulating CD8^+^ T cells (Figure 1A). Using gradient centrifugation, we isolated tumour‐infiltrating lymphocytes (TILs) and identified genuine CD8^+^ TRM in melanoma based on the indicated gating strategy (Figure 1B).

To validate the phenotype and function of CD8^+^ TRM, we enriched CD8^+^ TRM and other CD8^+^ T cells using a magnetic sorting strategy (Figure 1C) and confirmed the efficiency of the sorting system (Figure 1D). After reactivating CD8^+^ TRM and other CD8^+^ T cells in vitro, we used flow cytometry and ELISA to examine the expression of key effector molecules. We found that CD8^+^ TRM expressed higher levels of IFN‐γ (Figure 1E), PRF1 (Figure 1F) and GZMB (Figure 1G) compared to other CD8^+^ T cells. ELISA of the T‐cell culture supernatants further corroborated these findings (Figure 1H–J).

We then analysed human melanoma scRNA sequencing data of our previous study, performed dimensionality reduction and clustering of CD8^+^ T cells. Based on marker genes for distinct CD8^+^ T cell subsets and state scores for naïve, tissue‐resident, effector and exhausted CD8^+^ T cells (Figure S1D–G), we further subdivided CD8^+^ T cells into five subsets: CD8_Tn (naïve), CD8_Tem (effector memory), CD8_Trm (tissue‐resident), CD8_Tpex (progenitor exhausted) and CD8_Ttex (terminally exhausted; Figure 1K,L). We found that CD8^+^ TRM also expressed exhaustion‐related markers (Figure 1L). These findings were validated by flow cytometry (Figure 1M,N), suggesting that CD8^+^ TRM subsets may represent critical responders to ICB therapy.

### 3.2 CD103 is the specific marker for CD8^+^ TRM in melanoma

Studies have demonstrated that CD8**^+^** TRM exhibits tissue‐specific variations in their surface marker expression profiles.^19^ Among the well‐studied markers associated with the tissue‐resident phenotype are CD103^24^ and CD69,^25^ both of which facilitate the adhesion and retention of T lymphocytes within local tissues. However, conflicting conclusions have been reported regarding the expression levels of these relative markers on CD8^+^ TRM in different types of tissues. Therefore, we tested the ability of CD69 and CD103 to characterise the TRM subset in melanoma. Our flow cytometry results demonstrated that CD8^+^ TRM exhibited significantly higher CD103 expression level compared to other CD8^+^ T cells (Figure 2A). The finding was further supported by the result of scRNA‐seq data, which also revealed elevated CD103 expression in CD8^+^ TRM subset (Figure 2B). While, no obvious difference in CD69 expression was observed between CD8^+^ TRM and other CD8^+^ T cells (Figure 2B,C).

Through UMAP co‐localisation of single‐cell sequencing in melanoma, we found that the expression pattern of CD103 molecules is highly correlated with that of the more widely accepted marker molecules of the TRM subpopulation, such as ZNF683^26^, ^27^ and VCAM1^17^ (Figure S2A,B). The TRM score of the CD103^+^CD8^+^ subpopulation is significantly higher than CD103^−^CD8^+^ subpopulation (Figure S2C). This, in turn, further confirms that CD103 is one of the specific marker molecules of the TRM subpopulation in melanoma. Similar to the previously observed features of CD8^+^ TRM subsets, we found that CD8^+^CD103^+^ T cells exhibited higher levels of both exhaustion‐related markers (Figure 2D) and effector‐related molecules (Figure 2E) compared to other CD8^+^ T cells. Next, we investigated the expression of key molecules of the TRM within CD103^+^CD8^+^ cells and other CD8^+^ cells, further confirming that CD103^+^CD8^+^ T lymphocytes are bona fide CD8 TRM cells (Figure 2F). The immunofluorescence picture displayed a typical feature of CD8^+^ TRM, manifested as CD103 positivity (Figure 2G). These findings confirm that CD103 can serve as a specific marker for CD8^+^ TRM in the melanoma microenvironment.

### 3.3 CD103^+^CD8^+^ TRM presents enhanced proliferative potential

**FIGURE 3:**
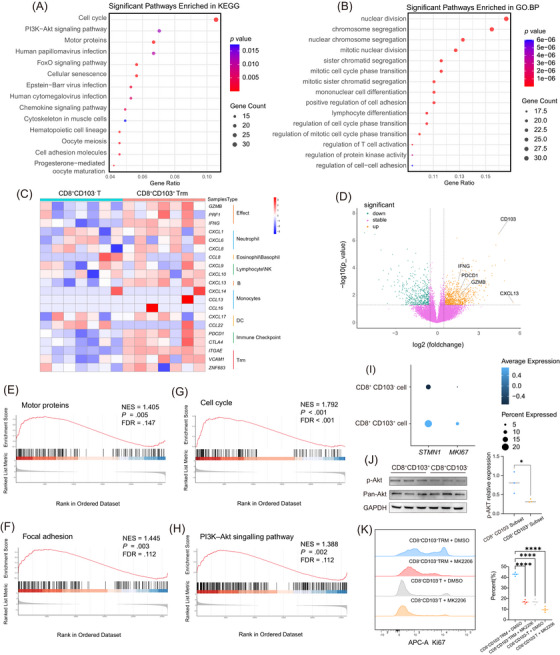
CD103^+^CD8^+^ tissue‐resident memory (TRM) presents enhanced proliferative potential. (A) KEGG enrichment analysis based on upregulated differentially expressed genes (DEGs) in CD103^+^CD8^+^ TRM cells. (B) GO.BP enrichment analysis based on upregulated DEGs in CD103^+^CD8^+^ TRM cells. (C) Heatmap of differential gene expression in the transcriptomes of CD103^+^CD8^+^ TRM cells and CD103^−^CD8^+^ cells. (D) Volcano plot of differential expression gene in the transcriptomes of CD103^+^CD8^+^ TRM cells and CD103^−^CD8^+^ cells. (E–H) Gene set enrichment analysis (GSEA) enrichment analysis of related pathways. (I) Single‐Cell sequencing analysis of expression of proliferation‐related molecules. (J) Western blot detection of activation of the PI3K/AKT pathway. (K) Flow cytometry detection of expression of the proliferation marker Ki‐67.

To further elucidate the anti‐tumour mechanisms of CD103^+^CD8^+^ TRM, we analysed bulk RNA sequencing data obtained from flow cytometry‐sorted CD103^+^CD8^+^ TRM cells and CD103^−^CD8^+^ circulating T lymphocytes. We first performed enrichment analyses on the upregulated DEGs in CD103^+^CD8^+^ TRM cells (Figure 3A,B). The enrichment results revealed that DEGs were associated with cell proliferation, cell cycle regulation, secretory function, chemokine/cytokine signalling and lymphocyte activation.

Based on these findings, we hypothesised that CD8^+^CD103^+^ TRM cells may possess a proliferative advantage and potentially participate in microenvironmental modulation through chemokine signalling. We therefore examined the differential expression of related genes. Notably, CD103^+^CD8^+^ TRM cells exhibited higher expression of markers relevant to immune checkpoint responses, in agreement with our prior single‐cell RNA‐seq and in vitro experimental results. Expression analysis of chemokines revealed that CD103^+^CD8^+^ TRM cells may play a central role in recruiting B cells, neutrophils and so on, with the B cell‐recruiting chemokine CXCL13^28^ showing the most prominent differential expression (Figure 3C,D).

Subsequent GSEA further demonstrated that CD103^+^CD8^+^ TRM cells were enriched in motor protein‐related pathways, indicating enhanced secretory capacity (Figure 3E), thus reinforcing their robust ability to secrete critical cytokines and effector molecules. Moreover, the significant enrichment of pathways associated with local adhesion supports the stable tissue residency phenotype of the CD103^+^CD8^+^ subset (Figure 3F). Together, these transcriptomic findings provide molecular evidence of the TRM identity of CD103^+^CD8^+^ TRM cells and underscore their potential in mounting anti‐tumour immune responses.

GSEA also validated the proliferative potential of CD103^+^CD8^+^ TRM cells (Figure 3G,H). This was corroborated by our scRNA‐seq data, which showed elevated proliferative activity in CD103^+^CD8^+^ TRM subsets (Figure 3I). Previous studies have proved that integrins can upregulate cell proliferation by activating the downstream PI3K/AKT signalling pathway^29^ and influence cell differentiation processes.^30^ As a key member of the integrin family, CD103 may likewise mediate downstream signalling to activate PI3K/AKT and support proliferation in CD103^+^CD8^+^ TRM cells.

Then, we performed Western blot assays to examine the activation status of the AKT signalling pathway in CD103^+^CD8^+^ TRM cells (Figure 3J). Furthermore, we assessed Ki67 expression—a marker of cell proliferation—via flow cytometry. We found that CD103^+^CD8^+^ TRM cells displayed significantly higher levels of Ki67 expression, which could be suppressed by an AKT inhibitor (Figure 3K). These findings revealed that CD103^+^CD8^+^ TRM cells exhibit a pronounced proliferative advantage, which is likely critical for sustaining robust anti‐tumour immune responses.

### 3.4 CD103^+^CD8^+^ TRM exhibits potent anti‐tumour activity

**FIGURE 4:**
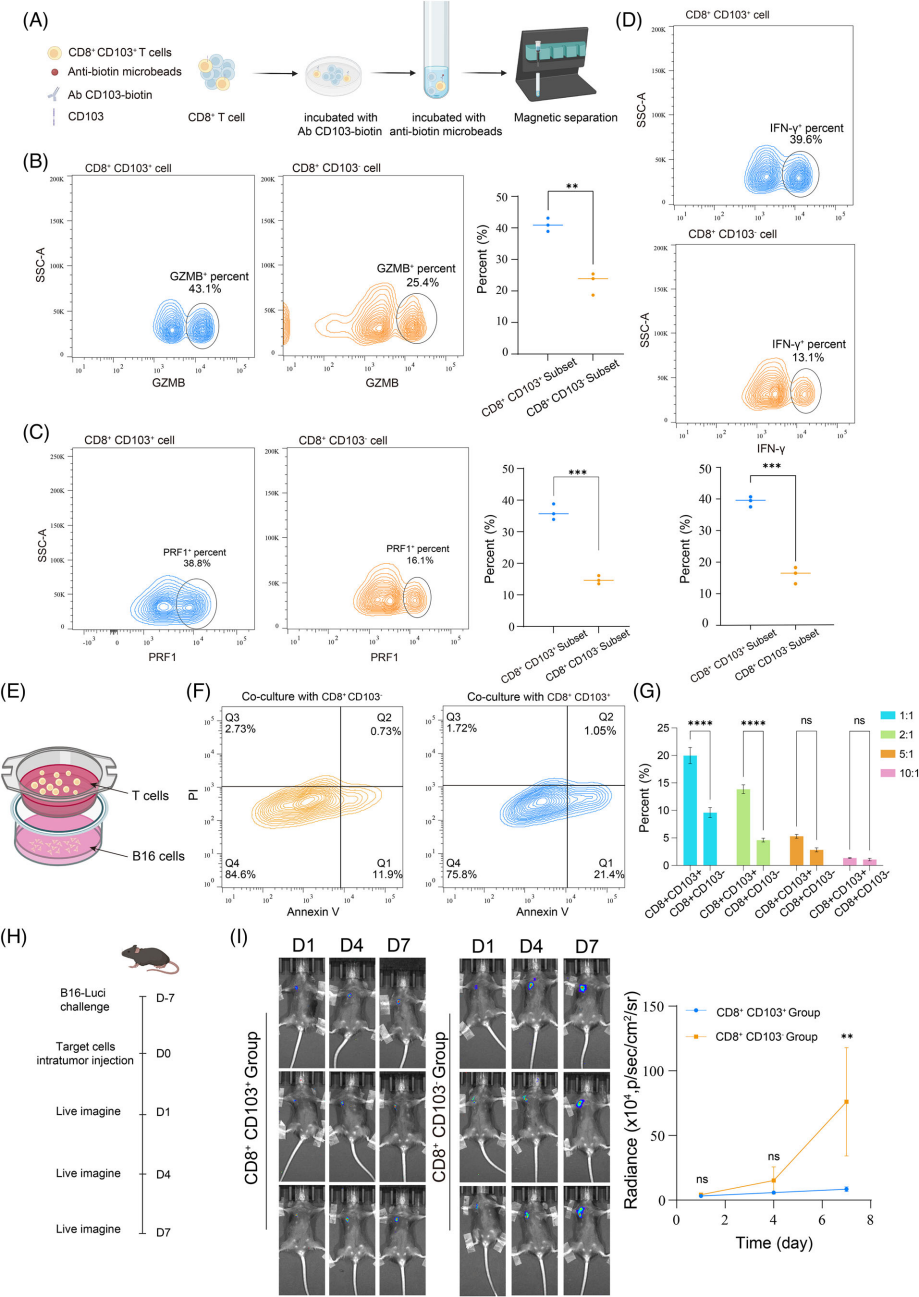
CD103^+^CD8^+^ tissue‐resident memory (TRM) exhibits potent anti‐tumour activity. (A) Magnetic sorting strategy diagram for CD103^+^CD8^+^ cells and CD103^−^CD8^+^ cells. (B–D) Flow cytometry detection of expression of effector‐related markers in CD103^+^CD8^+^ cells and CD103^−^CD8^+^ cells. (E) Schematic of the Transwell experiment: B16F10 melanoma cells were seeded in the lower chamber of the Transwell, while T cells (CD103^+^CD8^+^ cells or CD103^−^CD8^+^ cells) were seeded in the upper chamber. After 48 h, the apoptosis of melanoma cells co‐cultured with the two different T cells was analysed. (F) Under an effector‐to‐target (E:T) ratio of 1:1 (500 000 B16F10 cells co‐cultured with 500 000 T cells), apoptosis of B16F10 cells was assessed by flow cytometry. (G) The apoptosis of B16F10 cells was assessed by flow cytometry under various E:T ratios. (H, I) Schematic of the in vivo experiment: mice were subcutaneously injected with B16‐Luci cells (expressing luciferase). Seven days later, intratumourally adoptive transfer of T lymphocytes (CD103^+^CD8^+^ cells or CD103^−^CD8^+^ cells) was performed. Tumour growth was subsequently monitored through three rounds of bioluminescence imaging.

Based on the transcriptomic characteristics observed above, we hypothesised that CD8^+^CD103^+^ TRM possesses robust tumour‐killing capacity. Using a similar magnetic sorting strategy as described previously, we isolated CD103^+^CD8^+^ TRM cells and CD103^−^CD8^+^ T cell cells (Figure 4A). We analysed the expression of effector‐related molecules in these cells using flow cytometry, and observed that CD103^+^CD8^+^ TRM had higher expression levels of GZMB (Figure 4B), PRF1 (Figure 4C) and IFN‐γ (Figure 4D). Next, we conducted Transwell co‐culture experiments with these T cell subsets and B16F10 melanoma cells (Figure 4E). Flow cytometric analysis revealed that melanoma cells co‐cultured with CD103^+^CD8^+^ TRM showed a significantly higher proportion of apoptosis after 48 h (Figure 4F). Subsequently, co‐cultures were performed at different effector‐to‐target ratios, and it was observed that CD103^+^CD8^+^ TRM cells demonstrated a concentration‐dependent, superior cytotoxicity against tumour cells in vitro (Figure 4G). We then used lentiviral transduction to construct a luciferase‐expressing B16F10‐Luci cell line and conducted in vivo experiments (Figures 4H and S3A). These two T cell subsets were separately infused into tumour‐bearing mice, and tumour growth differences were observed. Notably, tumour growth in mice receiving CD103^+^CD8^+^ TRM was significantly suppressed compared to the other group (Figure 4I). Subsequently, we also observed in the lung metastasis model that the reinfusion of CD103^+^CD8^+^ TRM cells significantly inhibited tumour growth (Figure S3B). All these findings demonstrate that the CD103^+^CD8^+^ TRM possesses potent anti‐tumour capabilities and represents a key population responsible for tumour immune surveillance and cytotoxic activity.

### 3.5 The spatial distribution of CD103^+^CD8^+^ TRM cells and TLS is significantly correlated

Through our previous investigations of CD103^+^CD8^+^ TRM cells, we have discovered that, in addition to their inherent cytotoxic capabilities, these cells possess a robust secretory function, which may play a critical role in chemotaxis of immune cells and the improvement of the local microenvironment. Therefore, we initially conducted an immune infiltration analysis on the sequencing data of all melanoma patients from TCGA cohort.

We observed that in melanoma patient samples with high CD103^+^CD8^+^ TRM scores, the scores of immune effector cells, such as M1 macrophages, memory B lymphocytes, were significantly higher (Figure S4A). Gene expression correlation analysis of TCGA patient samples also revealed that the expression of the CD103 molecule was correlated with the scores of these cytotoxic effector cells (Figure S4B). Immunofluorescence staining and HALO spatial analysis of CD103^+^CD8^+^ TRM cells and B lymphocytes in melanoma revealed a spatial proximity between these two cell types (Figure S4C,D). ScRNA‐seq and flow cytometry analysis also revealed the high expression of the key chemokine CXCL13 in CD103^+^CD8^+^ TRM cells (Figure S4E,F). CXCL13 has been widely demonstrated^28^, ^31^, ^32^ to play a significant role in the recruitment of B lymphocytes, thus potentially representing a mechanism by which CD103^+^CD8^+^ TRM cells recruit B cells. What's more, we performed cell–cell communication analysis using the ‘CellChat’ algorithm to explore the expression of ligand–receptor pairs between CD103^+^CD8^+^ TRM cells, CD103^−^CD8^+^ T cells and other cell types. Notably, CD103^+^CD8^+^ TRM cells exhibited a higher number and diversity of predicted ligand–receptor interactions compared to CD103^−^CD8^+^ T cells, indicating a more active role in modulating the tumour microenvironment (Figure S4G).

**FIGURE 5:**
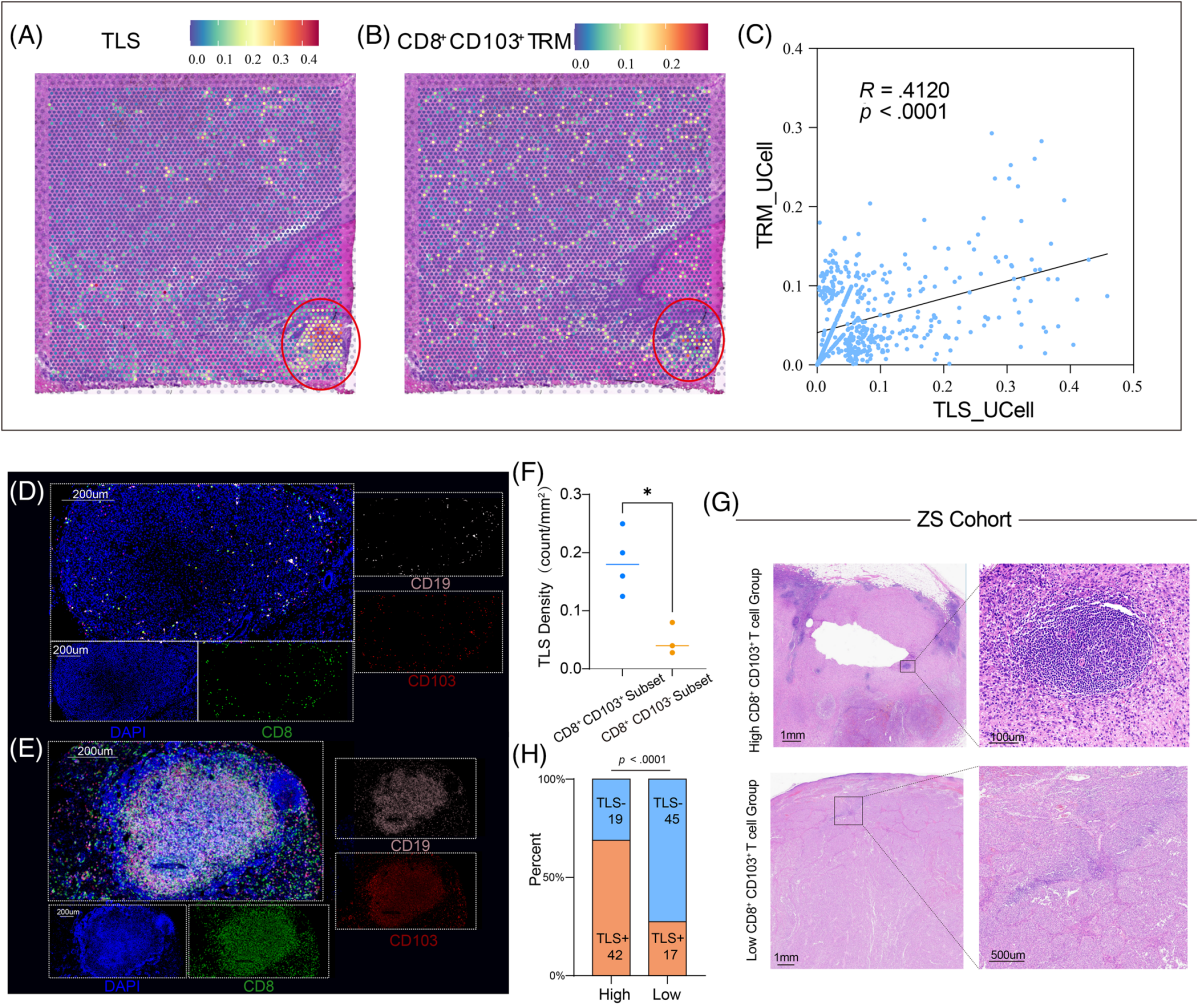
The spatial distribution of CD103^+^CD8^+^ tissue‐resident memory (TRM) cells and tertiary lymphoid structures (TLS) is significantly correlated. (A) Distribution of TLS in melanoma spatial transcriptomics samples; (B) Distribution of CD103^+^CD8^+^ TRM cells in melanoma spatial transcriptomics samples; (C) Correlation analysis between CD103^+^CD8^+^ TRM cells and TLS in melanoma spatial transcriptomics samples; (D–F) Representative images and comparison of TLS in tumours from groups infused with CD103^+^CD8^+^ TRM cells and CD103^−^CD8^+^ T cells; (G, H) Typical haematoxylin and eosin (H&E) staining images and comparison of the proportion of TLS‐positive tumours between groups with high and low CD103^+^CD8^+^ TRM cell counts.

The spatial proximity of T cells and B cells can form structures resembling TLS, which are considered important markers of immune therapy response.^32^ Together, we hypothesised that CD103^+^CD8^+^ TRM cells and TLS might also exhibit a spatial colocalisation relationship. Using spatial transcriptomic sequencing from our previous study,^33^ we analysed the spatial distribution relationship between various immune cells, CD103^+^CD8^+^ TRM cells and TLS (Figures S4H–K and 5A,B). We found that, compared with CD103^−^CD8^+^ T cells, CD103^+^CD8^+^ TRM cells exhibited a significant spatial correlation with TLS (Figures 5C and S4M). In vivo adoptive cell transfer experiments confirmed that the transfer of CD103^+^CD8^+^ TRM cells can influence the distribution of TLS (Figure 5D–F). We subsequently further confirmed in the Zhongshan Hospital cohort samples that samples with high CD103^+^CD8^+^ TRM density also had higher TLS density (Figure 5G,H). These findings suggested that the distribution of CD103^+^CD8^+^ TRM cells and TLS exhibits a significant spatial colocalisation relationship, which may be closely associated with patient prognosis and immune therapy response.

### 3.6 Higher intratumoural CD103^+^CD8^+^ TRM predicts better prognosis in melanoma

**FIGURE 6:**
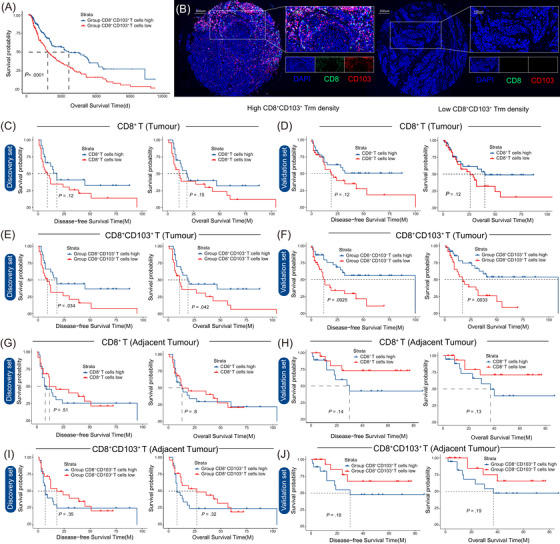
Higher intratumoural CD103^+^CD8^+^ tissue‐resident memory (TRM) predicts favourable prognosis in melanoma patients. (A) Survival analysis based on the CD8^+^CD103^+^ TRM gene signature in the TCGA cohort (*n* = 457). (B) Representative microscopic images of CD8 and CD103 expression patterns. Grouping of patients from the discovery and validation sets based on sample cell density (CD8^+^ T cells or CD8^+^CD103^+^ T cells): Patients with cell density above the median were classified into the high‐density group (High), while those below the median were classified into the low‐density group (Low). (C, D) Survival analysis of patients in both cohorts based on CD8^+^ T cell density in tumour tissue. (E, F) Survival analysis of patients in both cohorts based on CD8^+^CD103^+^ T cells density in tumour tissue. (G, H) Survival analysis of patients in both cohorts based on CD8^+^ T cells density in adjacent non‐tumour tissue. (I, J) Survival analysis of patients in both cohorts based on CD8^+^CD103^+^ T cells density in adjacent non‐tumour tissue.

Given the potent tumour‐killing ability of TRM, we further explored its prognostic significance in melanoma patients by analysing samples from the Zhongshan Hospital melanoma cohort and TCGA melanoma cohort. Initially, we explored the prognostic significance of the CD103^+^CD8^+^ TRM subset gene signature in melanoma patients within the TCGA cohort. We found that the gene signature of CD103^+^CD8^+^ TRM cells could significantly predict patient prognosis (Figure 6A).

Subsequently, we conducted further investigations in the Zhongshan Hospital melanoma cohort. Using the HALO pathology analysis system, we quantified the density of CD8^+^CD103^+^ TRM in tumour samples from different melanoma patients in this cohort. Representative immunofluorescence images of tumour tissues with high and low densities of CD8^+^CD103^+^ TRM were shown (Figure 6B).

Studies have reported that the density of tumour‐infiltrating CD8^+^ T cells is closely associated with the prognosis of melanoma patients,^34^, ^35^ and the distribution of CD8^+^ T lymphocyte density differs between cancerous tissues and peritumoural invasive tissues.^36^ Therefore, we first performed survival analyses for patients in the high‐ and low‐density CD8^+^ T cell groups. However, neither the discovery cohort nor the validation cohort showed statistically significant differences in overall survival (OS) or disease‐free survival (DFS) between these two groups (Figure 6C,D). In contrast, survival analyses of patients stratified by CD103^+^CD8^+^ TRM density revealed significant correlations between higher densities of these cells and longer OS and DFS, a finding validated in both the discovery and validation cohorts (Figure 6E,F). To further explore whether these correlations were specific to tumour tissues, we grouped patients based on the density of CD103^+^CD8^+^ TRM or CD8^+^ T cells in matched adjacent non‐tumour samples and performed survival analyses. Interestingly, no significant association of patient survival times was observed between CD8^+^CD103^+^ TRM or CD8^+^ T cell density in adjacent non‐tumour tissues (Figure 6G–J). This finding suggested that CD103^+^CD8^+^ TRM likely play a critical anti‐tumour role within the tumour microenvironment, rather than in adjacent non‐tumour regions. In summary, the high density of CD103^+^CD8^+^ TRM in tumour tissues is a strong indicator of favourable prognosis in melanoma patients. We further conducted univariate and multivariate analyses to assess the prognostic significance of CD103^+^CD8^+^ TRM cell density along with other potential confounding variables, which confirmed the value of CD103^+^CD8^+^ TRM cells as an independent predictor of melanoma prognosis (Table 2).

**TABLE 2:** Univariate and multivariate prognostic analyses related to overall survival (OS) and disease‐free survival (DFS).

	Overall survival						Disease‐free survival					
	Discovery set (*n* = 49)			Validation set (*n* = 74)			Discovery set (*n* = 49)			Validation set (*n* = 74)		
	Univariate *p* value	Multivariate *p* value	Multivariate HR (95%CI)	Univariate *p* value	Multivariate *p* value	Multivariate HR (95%CI)	Univariate *p* value	Multivariate *p* value	Multivariate HR (95%CI)	Univariate *p* value	Multivariate *p* value	Multivariate HR (95%CI)
**Age, years**	.6254	NA	NA	.1331	NA	NA	.3662	NA	NA	.1011	NA	NA
(A0r vs. <60)												
**Gender**	.2553	NA	NA	.8875	NA	NA	.7626	NA	NA	.6281	NA	NA
(male vs. female)												
**Anatomic site**	.0695	NA	NA	.2442	NA	NA	.0366	.4193	.8128 (.4914–1.3442)	.1679	NA	NA
(acral vs. trunk vs. other)												
**Histologic type**	.1542	NA	NA	.0525	NA	NA	.0282	.0330	.6478 (.4346–.9657)	.0671	NA	NA
(superficial spreading vs. nodular vs. acral vs. lentigo maligna)												
**Breslow depth (mm)**	.3592	NA	NA	.4712	NA	NA	.8625	NA	NA	.1900	NA	NA
(>2 vs. ≤2)												
**Clark level**	.1235	NA	NA	.0859	NA	NA	.0354	.2738	1.5975 (.6904–3.6966)	.1306	NA	NA
(IV966vs. I–III)												
**Ulceration**	.7368	NA	NA	.0394	.1649	.2399 (.0320–1.7984)	.5311	NA	NA	.0183	.1802	.2515 (.0334–1.8933)
(present vs. absent)												
**Lymph nodes metastasis**	.0621	NA	NA	.0847	NA	NA	.1201	NA	NA	.0807	NA	NA
(yes vs. no)												
**Distant metastasis**	.2385	NA	NA	.2000	NA	NA	.2495	NA	NA	.2345	NA	NA
(yes vs. no)												
**Clinical**	.0219	.0086	2.7775 (1.2956–5.9544)	.0152	.0005	3.5119 (1.7270–7.1414)	.0337	.2136	1.7486 (.7248–4.2186)	.0181	.0036	3.0076 (1.4343–6.3070)
(III–V vs. I–II)												
**CD8^+^CD103^+^T density**	.0181	.0121	.4076 (.2022–.8216)	.0234	.0335	.4723 (.2365–.9431)	.0137	.0030	.2839 (.1235–.6527)	.0087	.0435	.4745 (.2301–.9784)
(high vs. low)												
**CD8^+^T density**	.1279	NA	NA	.3015	NA	NA	.0924	NA	NA	.2055	NA	NA
(high vs. low)												

Abbreviations: HR, hazard ratio; CI, confidence interval; NA, not available. *p* < .05 was considered significant.

### 3.7 CD103^+^CD8^+^ TRM predicts response to ICB therapy in melanoma

ICB therapy has revolutionised the treatment of melanoma. However, some patients still fail to benefit from ICB treatment and are at risk for immune‐related adverse events. Therefore, identifying biomarkers for predicting ICB therapy response in melanoma is urgently needed. TLS have been shown to be a critical structural basis for ICB response,^37^ and we found that CD8^+^CD103^+^ TRM can promote TLS formation. Consequently, we explored the potential of CD103^+^CD8^+^ TRM as a biomarker for predicting ICB treatment response in melanoma.

**FIGURE 7:**
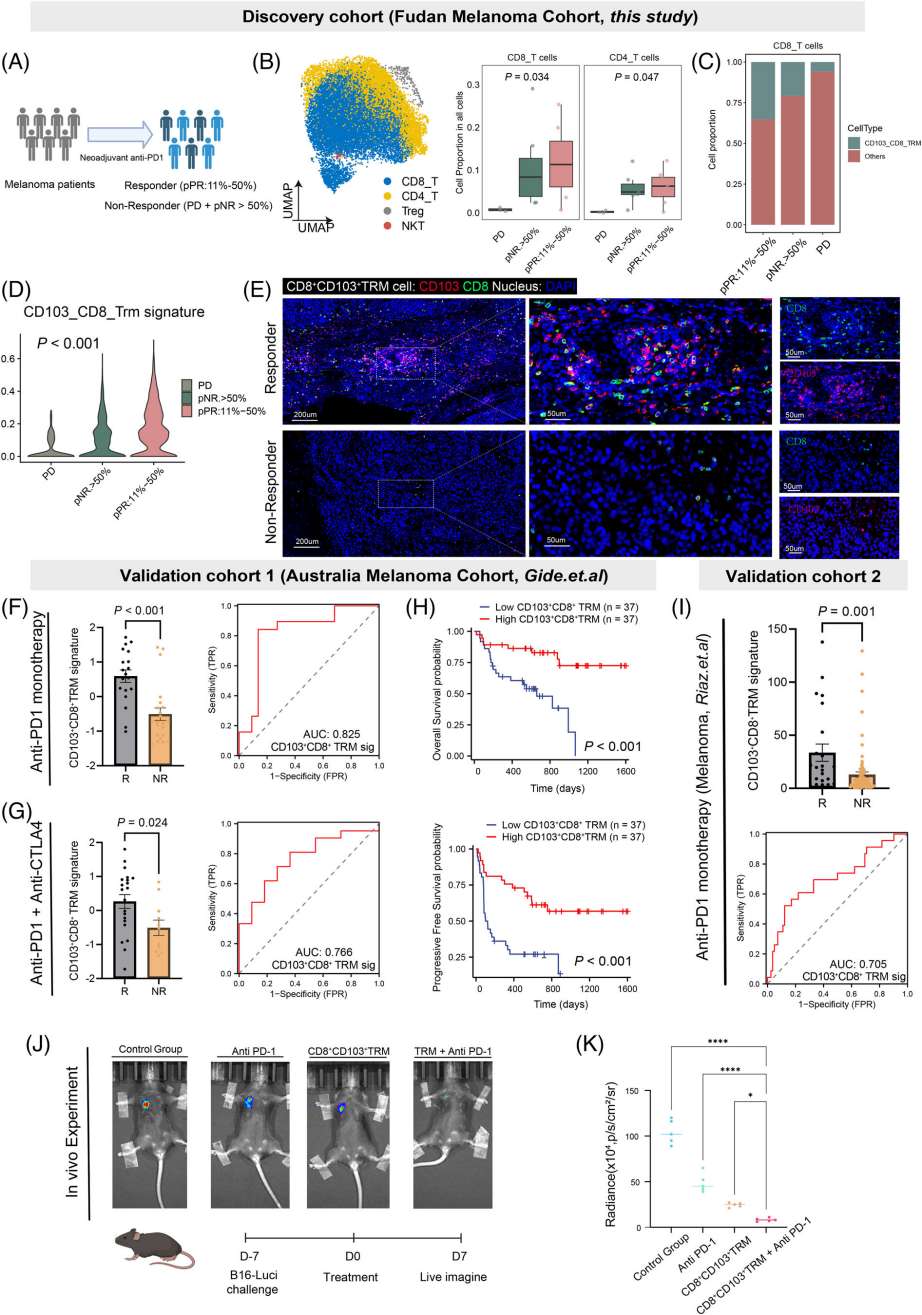
CD103^+^CD8^+^ tissue‐resident memory (TRM) predicts response to immune checkpoint blockade (ICB) therapy in melanoma. (A) Clinical feature heatmap for the discovery cohort. (B) UMAP plot of T‐cell subsets and their proportions in tumours with different responses to immunotherapy. (C) Proportion of CD103^+^CD8^+^ TRM in CD8^+^ T cells infiltrating tumours with different responses to immunotherapy. (D) Comparison of CD103^+^CD8^+^ TRM signature in CD8^+^ T cells infiltrating tumours with different responses to immunotherapy. (E) Representative staining images of CD103^+^CD8^+^ TRM in tumours with different responses to immunotherapy and comparison. (F) Comparison of CD103^+^CD8^+^ TRM signature in PD‐1 therapy responders and non‐responders and ROC curve for predicting response. (G) Comparison of CD103^+^CD8^+^ TRM signature in PD‐1 therapy combined with CTLA‐4 therapy responders and non‐responders and ROC curve for predicting response. (H) Impact of CD103^+^CD8^+^ TRM on overall survival (OS) and PFS in immunotherapy responders. (I) Comparison of CD103^+^CD8^+^ TRM signature in PD‐1 therapy responders and non‐responders and ROC curve for predicting response. (J, K) In vivo experiments (bioluminescence imaging): Control group, anti‐PD‐1 treatment group, CD103^+^CD8^+^ TRM treatment group and combination treatment group.

First, we used the single‐nucleus sequencing dataset from melanoma patients receiving neoadjuvant immunotherapy as the discovery cohort.^38^ This dataset included 19 melanoma samples from patients who experienced progression disease (PD), stable disease (SD, defined as residual tumour cells >50%, pNR > 50%) or partial response (PR, defined as residual tumour cells ≤50%, pNR 11%–50%) after neoadjuvant therapy (Figure 7A). We observed that tumours with PR had a significantly higher proportion of CD4^+^ T cells and CD8^+^ T cells compared to PD and SD tumours (Figure 7B). Importantly, we found that CD103^+^CD8^+^ TRM represented a higher proportion of CD8^+^ T cells in PR patients, while PD tumours contained almost no CD103^+^CD8^+^ TRM (Figure 7C). Furthermore, the CD103^+^CD8^+^ TRM signature was higher in PR tumours compared to PD and SD tumours (Figure 7D). Immunofluorescence staining also confirmed the enrichment of CD103^+^CD8^+^ TRM in the tumours of PR patients (Figures 7E and S5A). These results suggested that CD103^+^CD8^+^ TRM were associated with melanoma patients' response to ICB therapy.

To further validate the predictive value of CD103^+^CD8^+^ TRM for response to ICB therapy, we used two additional external clinical cohorts. Consistent with the findings in the discovery cohort, CD103^+^CD8^+^ TRM signature was significantly enriched in patients who responded to PD‐1 monoclonal antibody treatment, and it showed excellent predictive value for the efficacy of PD‐1 monotherapy (Figure 7F).^39^, ^40^ Interestingly, we discovered that the CD103^+^CD8^+^ TRM signature not only predicted the efficacy of PD‐1 monotherapy but also had predictive value for the efficacy of combined PD‐1 and CTLA‐4 antibody immunotherapy (Figure 7G). Furthermore, the CD103^+^CD8^+^ TRM signature was able to distinguish the prognosis of patients receiving ICB therapy. Patients with the high CD103^+^CD8^+^ TRM signature had better prognosis, while those with the low signature had poorer outcomes (Figure 7H). Another independent melanoma immunotherapy cohort also confirmed that the CD103^+^CD8^+^ TRM signature had excellent predictive value for ICB treatment efficacy (Figure 7I). Then, we compared the predictive performance of the CD103^+^CD8^+^ TRM signature with PD‐L1 expression in these immunotherapy cohorts. The results showed that in validation cohort 1 (Figure S5B,C), the predictive performance of this signature was comparable to that of PD‐L1 expression, while in validation cohort 2 (Figure S5D), it was higher than PD‐L1 expression. These results from independent clinical cohorts indicate that CD103^+^CD8^+^ TRM was significantly enriched in immunotherapy responders and hold predictive value for treatment response.

Then, we employed a preclinical animal model and administered a combination of PD‐1 inhibitor treatment and adoptive transfer of CD103^+^CD8^+^ TRM cells. We found that in mice treated with the combination of CD103^+^CD8^+^ TRM cells and PD‐1 inhibitor, tumour growth was significantly inhibited, thereby confirming that CD103^+^CD8^+^ TRM cells can potentiate the therapeutic effects of immunotherapy (Figure 7J,K).

## 4 DISCUSSION

The transformative success of ICB therapy in the treatment of solid tumours represents a landmark achievement in oncology,^41^, ^42^, ^43^ underscoring the pivotal importance of dissecting the tumour immune microenvironment and modulating immune cell states as an effective therapeutic strategy.^44^ Among the cellular subsets regulated by ICB, TILs play a central role in immune surveillance and cytotoxic responses within the immune microenvironment,^45^, ^46^ drawing significant attention and becoming a focal point of immunological research. Advancements in TIL studies have unveiled their inherent heterogeneity and the distinct roles that different TIL phenotypes play in adaptive immune responses.^47^

Emerging evidence highlights the indispensable function of TILs with a TRM phenotype in orchestrating immune surveillance within local microenvironments during chronic infections^48^ and tumour progression.^49^ The identification of surface‐specific marker molecules for TRM subsets is of critical importance for their isolation, functional characterisation and clinical translation. Although commonly used TRM surface markers such as CD103 and CD69 have been applied in studies of other tumour types, previous research has shown that the expression of TRM‐associated markers varies across tissues and exhibits a degree of tissue specificity. To date, no study has systematically evaluated the specificity of these commonly used surface markers in melanoma‐derived TRM subsets. We therefore considered it essential to address this question as a first step. High‐dimensional approaches such as mass cytometry and scRNA‐seq may introduce excessive cellular heterogeneity, potentially leading to false‐positive findings when attempting to define TRM characteristics. Drawing from methodologies in prior studies, we established an adoptive cell transfer mouse model to rigorously define bona fide TRM subsets in the melanoma microenvironment based on two defining phenotypes: tissue residency and memory. By integrating in vitro experiments with single‐cell transcriptomic analysis, we identified CD103 as a specific and reliable marker for CD8^+^ TRM subsets within the melanoma microenvironment. This finding provides a robust molecular basis for downstream functional and translational research on melanoma‐infiltrating TRM cells. Through in vitro and in vivo experiments, we found the therapeutic potential of these cells, marking them as key players in melanoma immunity. Our current study revealed spatial colocalisation between the CD103^+^CD8^+^ TRM subset and TLS. However, due to the limitations of our experimental design, we cannot rule out the involvement of other cell subsets—such as follicular helper T (TFH) cells^50^—in this process. As a result, a direct causal relationship between CD103^+^CD8^+^ TRM cells and TLS formation cannot be definitively established at this stage. We plan to further investigate this question using gene‐deficient mouse models in future experiments. In addition, previous studies have reported that the robust immune pressure exerted by TRM cells within the local tumour microenvironment may drive malignant cells to downregulate MHC molecule expression, thereby facilitating immune evasion.^51^ Therefore, the precise role of CD103^+^CD8^+^ TRM cells in shaping the tumour microenvironment requires further exploration. Some researchers have refined the phenotypic definition of CD103^+^CD8^+^ TRM cells by incorporating additional molecular markers such as CD39,^52^, ^53^, ^54^ an effector marker, and CD49a, a molecule associated with adhesion.^55^, ^56^ Co‐expression of these markers with CD103 may enhance both the functional capacity and prognostic value of this TRM subset. These findings suggest that a certain degree of heterogeneity and differentiation may exist within the CD103^+^CD8^+^ TRM subset itself, indicating that our current classification of this population as a single, uniform entity may be an oversimplification. Some studies have suggested that TGFβ can enhance the cytotoxic effects of CD103^+^ cytotoxic T lymphocytes (CTLs) and acts as an upstream regulatory factor for CD103^+^ CTLs.^57^, ^58^ In future research work, we will deeply explore the evolution, metabolic dynamics and functional diversification within the CD103^+^CD8^+^ TRM cell population, thereby clarifying the specific mechanism of this subset playing a key regulatory role in the tumour microenvironment. Moreover, emerging computational approaches such as machine learning have recently been applied to CD103^+^CD8^+^ TRM studies, further substantiating the prognostic relevance of this subset in cancer.^59^

CD8^+^ T lymphocytes are a key cell population in cellular immunity and have traditionally been defined as cytotoxic T cells. However, with growing insights into T cell biology, it has become evident that both CD4^+^ and CD8^+^ T lymphocytes comprise diverse subsets, each expressing distinct surface markers and performing specialised functions. Moreover, during T cell activation, surface molecules such as PD‐1 and CTLA‐4—traditionally regarded as markers of exhaustion—are often upregulated. Yet, the upregulation of these markers does not necessarily indicate full functional exhaustion. Current research suggests that T cell differentiation and death occur through a gradual process, with various intermediate or transitional states. These intermediate cells may still retain essential effector functions and are likely the critical targets reactivated by ICB therapies. This may explain why, in our findings, overall density of CD8^+^ T cells did not strongly predict patient prognosis. We have verified the prognostic value of genetic characteristics associated with the CD103 + CD8 + TRM subgroup in the published TCGA cohort. In our melanoma patient cohort, we observed a significant correlation between the higher distribution of CD8^+^CD103^+^ TRM cells within the tumour microenvironment and favourable patient outcomes. Moreover, analyses of immunotherapy cohorts confirmed the strong predictive value of CD8^+^CD103^+^ TRM genetic signatures for immunotherapy responsiveness. Due to the limited availability of clinical specimens, we were unable to extend our conclusions to additional cancer types. Conducting multi‐centre clinical studies will be a key focus of our future research efforts.

TIL‐based therapy has become as a promising way for therapy,^60^ with the FDA recently approving the first TIL product for clinical application. Enhancing the efficacy and response rates of TIL therapy remains a critical challenge. Our findings suggest that isolating and enriching key anti‐tumour subsets, such as CD103^+^CD8^+^ TRM cells, represents a promising strategy to optimise this therapeutic approach. Our study demonstrated that adoptive transfer of the CD103^+^CD8^+^ TRM subset enhances sensitivity to ICB therapy, further confirming the translational potential of this cell population.

Our study delves into the anti‐tumour mechanisms and therapeutic and prognostic value of CD8^+^CD103^+^ TRM cells within the melanoma microenvironment. We establish these cells as a critical subset, serving as powerful predictors of favourable prognosis and immunotherapy responsiveness. These findings lay a solid foundation for future research aimed at improving TIL‐based therapies to achieve more superior clinical treatment results.

Additionally, further investigation is warranted into the interplay between the defining phenotypes of TRM cells—memory and tissue residency. For example, whether the upregulation of residency‐associated molecules facilitates TRM retention in local tissues, thereby increasing interactions with tissue‐specific antigens and promoting memory phenotype development, remains an open question. Moreover, the differentiation trajectory and transcriptional regulators governing CD8^+^CD103^+^ TRM cells represent crucial areas for future exploration.

As key players in adaptive immunity, T cells represent one of the immune system's most potent cytotoxic forces. The identification of the prognostic significance of the CD8^+^CD103^+^ TRM cells not only enhance our understanding of TIL heterogeneity but also holds transformative potential for improving the efficacy of melanoma immunotherapy and adoptive cell therapies. This discovery underscores the critical role of precise immune modulation in advancing TIL‐based treatments, paving the way for a new era of precision oncology.

## AUTHOR CONTRIBUTIONS

Tianyi Zhang, Yuan Gao, Zhi Pang and Lu Wang are responsible for conducting research and design work. Tianyi Zhang, Junquan Song, Lili Lu and Yinlam Li conducted relevant experiments in vitro and in vivo. Kangjie Shen, Jiangying Xuan and Yang Yang analysed and processed the data. Zixu Gao and Chenlu Wei completed the analysis of clinical data. Qianrong Hu, Yingyong Hou, Yuhong Zhou and Rongkui Luo were subjected to multiple immunohistochemical (mIHC) experimental procedures. Tianyi Zhang, Shaoluan Zheng, Yuhong Zhou, Jianying Gu and Chuanyuan Wei wrote the specific content of the manuscript. All the authors have read and approved the final manuscript content. The order of signatures of co‐first authors is determined based on their respective contribution levels.

## CONFLICT OF INTEREST STATEMENT

The authors declare no conflicts of interest.

## ETHICS STATEMENT

This study was approved by the Biomedical Research Ethics Committee of Zhongshan Hospital (Approval No. 2023‐036) and the Institutional Animal Care and Use Committee (IACUC) of Zhongshan Hospital (Approval No. 2023‐293).

## Supporting information



Figure S1 CD8^+^ tissue‐resident memory (TRM) exhibits potent tumour‐killing role. (A) Construction of the B16F10‐ova cell line (through lentiviral transfection with GFP tag). (B) Negative selection strategy for CD8^+^ T lymphocytes: Step 1: After digestion of the spleen or melanoma tissue, the cell suspension is filtered. Step 2: After centrifugation, obtain: (a) Supernatant; (b) Cell pellet. Step 3: Resuspend the cell pellet with Ficoll (1.084), (a) 1640 culture medium; (b) Cell suspension resuspended in Ficoll; (c) Fresh Ficoll. Step 4: Centrifuge at 800 × *g*, 25°C, slow acceleration and deceleration for 30 min. Step 5: After centrifugation, obtain mononuclear cells, (a) 1640 culture medium; (b) Mononuclear cells; (c) Ficoll; (d) Tumour cells, red blood cells and other high‐density cells precipitate. Step 6: Aspirate mononuclear cells. Step 7: Perform negative selection to obtain T cells from the mononuclear cells (Cat#11413D, Invitrogen, Cat#12303D, Invitrogen). Step 9: Use magnetic beads to deplete CD4^+^ T lymphocytes to obtain CD8^+^ T lymphocytes via negative selection (Cat#11445D, Invitrogen, Cat#12303D, Invitrogen). (C) Efficiency of the sorting strategy; (D–G) Scoring of CD8^+^ T lymphocyte‐related phenotypes.

Figure S2 CD103 is the specific marker for CD8^+^ tissue‐resident memory (TRM) in melanoma. (A, B) Single‐cell RNA sequencing analysis revealed uniform manifold approximation and projection (UMAP)‐based spatial colocalisation of CD103 with VCAM1 and ZNF683 within the CD8^+^ T lymphocyte population; (C) The TRM gene signature score in the CD103^+^CD8^+^ TRM subpopulation and the CD103^−^CD8^+^ T cell subpopulation.

Figure S3 CD103^+^CD8^+^ tissue‐resident memory (TRM) exhibits potent anti‐tumour activity. (A) Construction of the B16F10‐Luci cell line (through lentiviral transfection with GFP tag); (B) After establishing the lung metastasis model, adoptive cell animal experiments were conducted by reinfusing different cell subsets (CD103^+^CD8^+^ TRM subpopulation and CD103^−^CD8^+^ T cells). Subsequently, detection was performed using bioluminescence imaging.

Figure S4 The spatial distribution of CD103^+^CD8^+^ tissue‐resident memory (TRM) cells and tertiary lymphoid structures (TLS) is significantly correlated. (A) Immune infiltration analysis of melanoma samples in the TCGA cohort; (B) Correlation analysis between ITGAE expression levels and scores of different immune subsets in the sequencing data of TCGA melanoma samples; (C, D) Co‐localisation of the spatial distribution of CD103^+^CD8^+^ TRM cells and B lymphocytes; (E) Single‐cell sequencing analysis of CXCL13 expression in CD103^+^CD8^+^ TRM cells and CD103^−^CD8^+^ cells; (F) Flow cytometry detection of CXCL13 in CD103^+^CD8^+^ TRM cells and CD103^−^CD8^+^ cells; (G) Analysis of ligand‐receptor pairs of the CD103^+^CD8^+^ TRM subpopulation and CD103^−^CD8^+^ T cells; (H–K) Spatial transcriptomics analysis of relevant immune subsets; (L) The corresponding haematoxylin and eosin (H&E) images of the spatial transcriptomics data; (M) Spatial transcriptomics analysis of CD103^−^CD8^+^ T cell distribution.

Figure S5 CD103^+^CD8^+^ tissue‐resident memory (TRM) predicts response to immune checkpoint blockade (ICB) therapy in melanoma. (A) Statistical comparison of CD103^+^CD8^+^ TRM in tumours with different responses to immunotherapy. (B) ROC curve assessing the predictive value of PD‐L1 expression in the Gide cohort (PD‐1 therapy combined with CTLA‐4 therapy). (C) ROC curve assessing the predictive value of PD‐L1 expression in the Gide cohort (PD‐1 monotherapy). (D) ROC curve assessing the predictive value of PD‐L1 expression in the Riaz cohort (PD‐1 monotherapy).

## Data Availability

Raw and processed bulk‐RNAseq data of this study can be obtained from Gene Expression Omnibus (GEO) with an accession number of GEO: GSE194383. Any additional information required to reanalyse the data reported in this article is available from the lead contact upon request.
