# Supplementary figures and images for: CD103+CD8+ tissue‐resident memory T lymphocytes of melanoma boost anti‐tumour immunity and predict immunotherapy outcomes
Source: Clin Transl Med. 2025 Sep 23;15(9):e70464. doi: 10.1002/ctm2.70464 (PMC12456097; doi:10.1002/ctm2.70464)

A

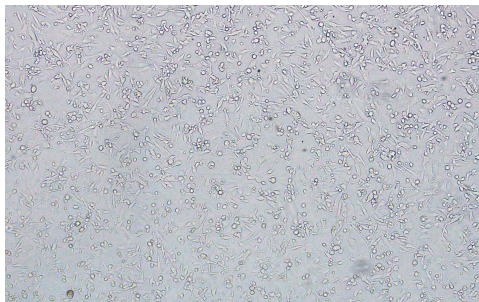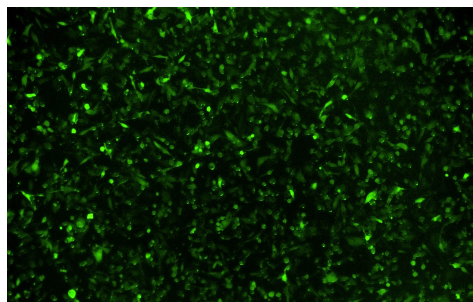

B

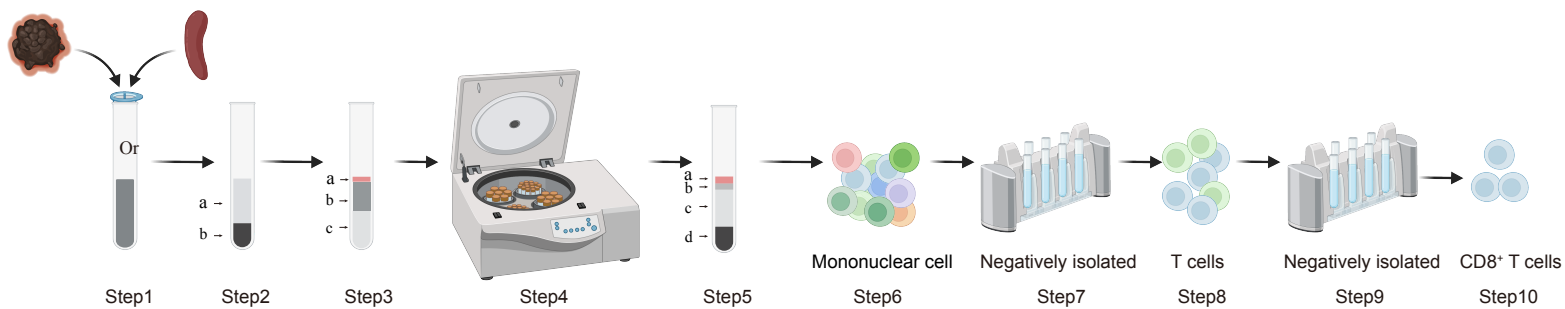

C

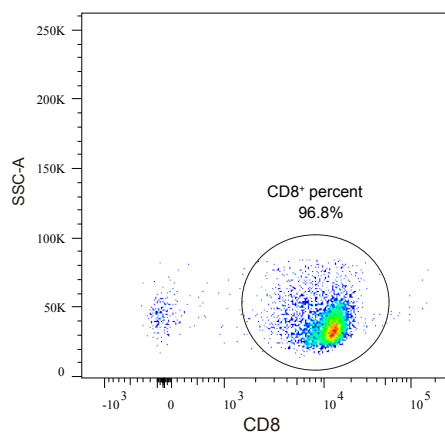

D

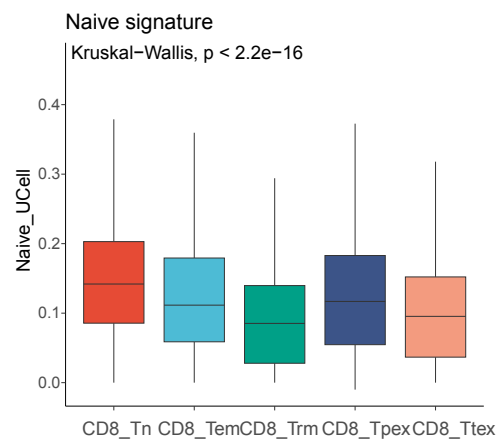

E

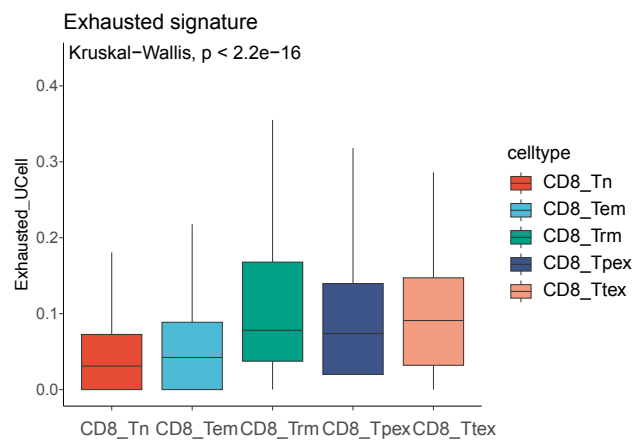

F

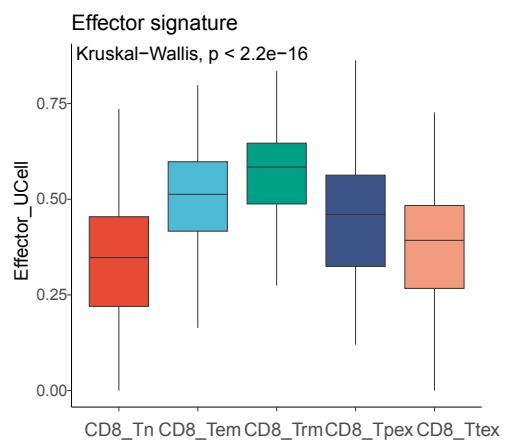

G

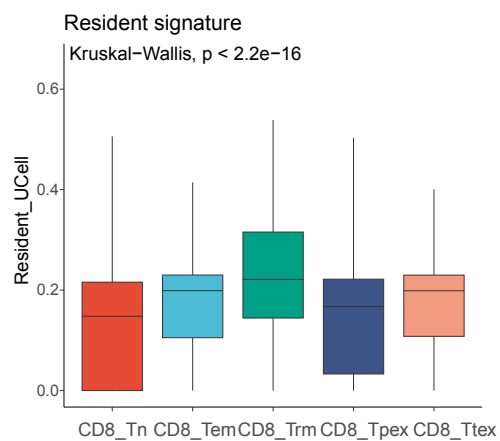

Supplement: Supplementary file 1 — Figure S1 CD8+ tissue‐resident memory (TRM) exhibits potent tumour‐killing role. (A) Construction of the B16F10‐ova cell line (through lentiviral transfection with GFP tag). (B) Negative selection strategy for CD8+ T lymphocytes: Step 1: After digestion of the spleen or melanoma tissue, the cell suspension is filtered. Step 2: After centrifugation, obtain: (a) Supernatant; (b) Cell pellet. Step 3: Resuspend the cell pellet with Ficoll (1.084), (a) 1640 culture medium; (b) Cell suspension resuspended in Ficoll; (c) Fresh Ficoll. Step 4: Centrifuge at 800 × g, 25°C, slow acceleration and deceleration for 30 min. Step 5: After centrifugation, obtain mononuclear cells, (a) 1640 culture medium; (b) Mononuclear cells; (c) Ficoll; (d) Tumour cells, red blood cells and other high‐density cells precipitate. Step 6: Aspirate mononuclear cells. Step 7: Perform negative selection to obtain T cells from the mononuclear cells (Cat#11413D, Invitrogen, Cat#12303D, Invitrogen). Step 9: Use magnetic beads to deplete CD4+ T lymphocytes to obtain CD8+ T lymphocytes via negative selection (Cat#11445D, Invitrogen, Cat#12303D, Invitrogen). (C) Efficiency of the sorting strategy; (D–G) Scoring of CD8+ T lymphocyte‐related phenotypes. [file CTM2-15-e70464-s001.pdf]

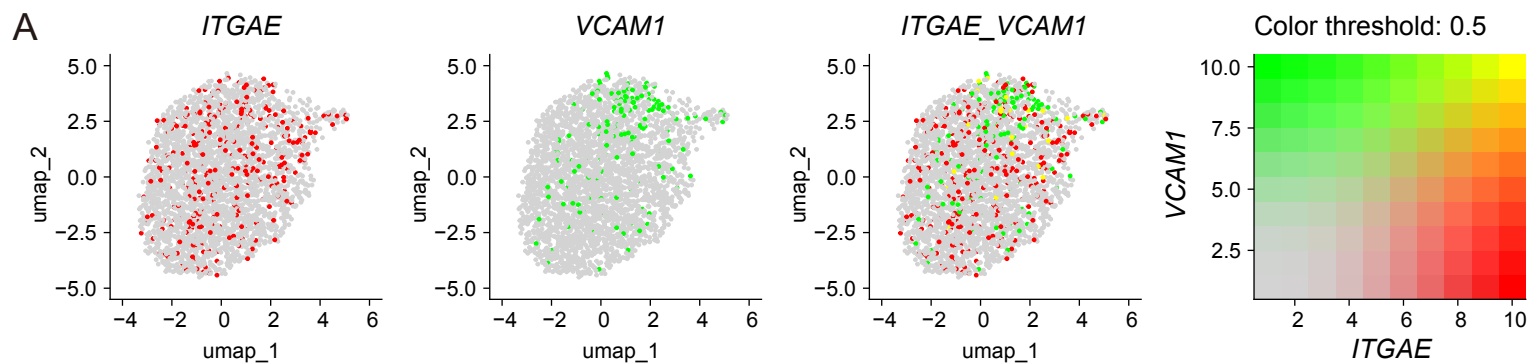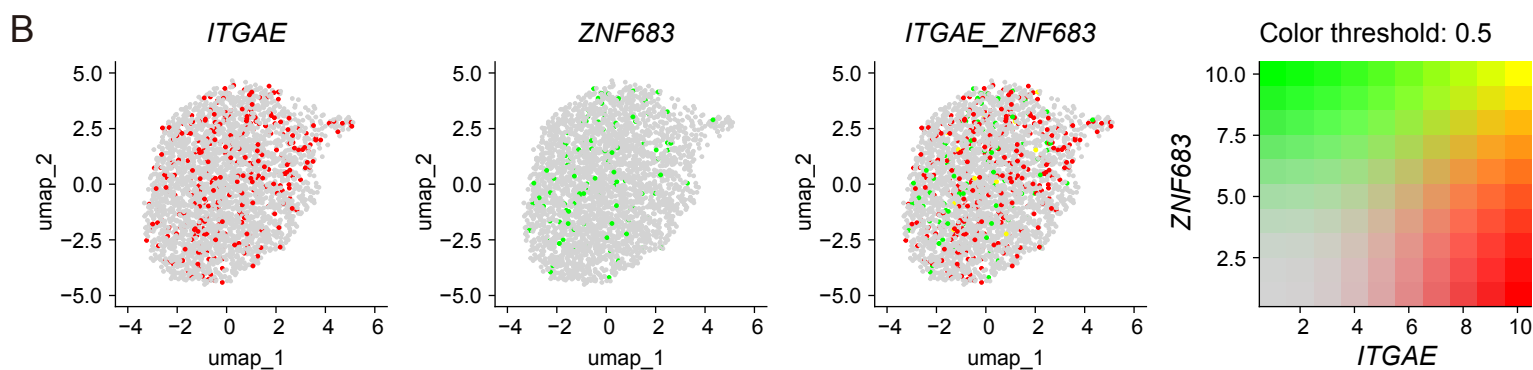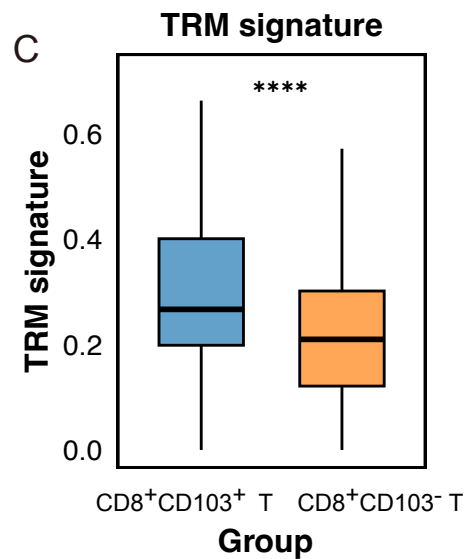

Supplement: Supplementary file 2 — Figure S2 CD103 is the specific marker for CD8+ tissue‐resident memory (TRM) in melanoma. (A, B) Single‐cell RNA sequencing analysis revealed uniform manifold approximation and projection (UMAP)‐based spatial colocalisation of CD103 with VCAM1 and ZNF683 within the CD8+ T lymphocyte population; (C) The TRM gene signature score in the CD103+CD8+ TRM subpopulation and the CD103−CD8+ T cell subpopulation. [file CTM2-15-e70464-s004.pdf]

A

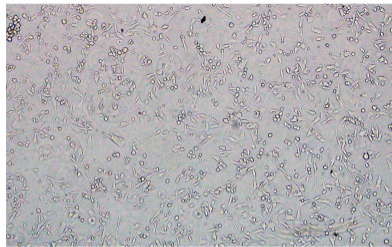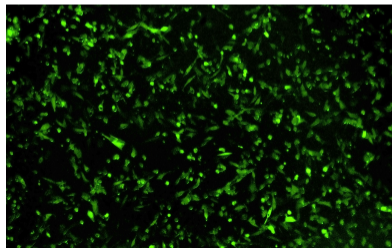

B

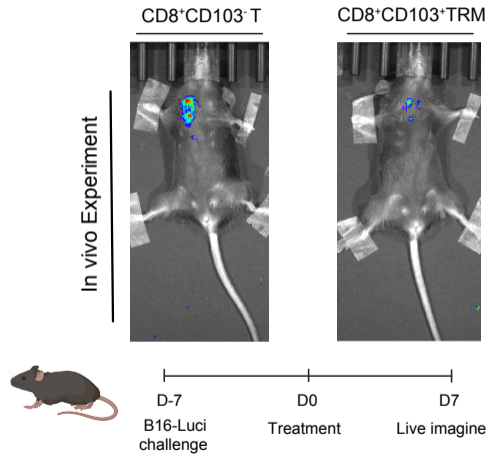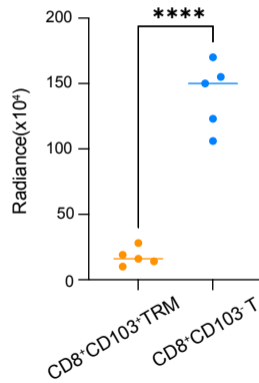

Supplement: Supplementary file 3 — Figure S3 CD103+CD8+ tissue‐resident memory (TRM) exhibits potent anti‐tumour activity. (A) Construction of the B16F10‐Luci cell line (through lentiviral transfection with GFP tag); (B) After establishing the lung metastasis model, adoptive cell animal experiments were conducted by reinfusing different cell subsets (CD103+CD8+ TRM subpopulation and CD103−CD8+ T cells). Subsequently, detection was performed using bioluminescence imaging. [file CTM2-15-e70464-s003.pdf]

**A**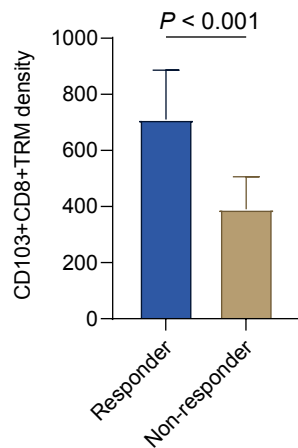

**Validation cohort 1 (Australia Melanoma Cohort, *Gide.et.al*)**

**B**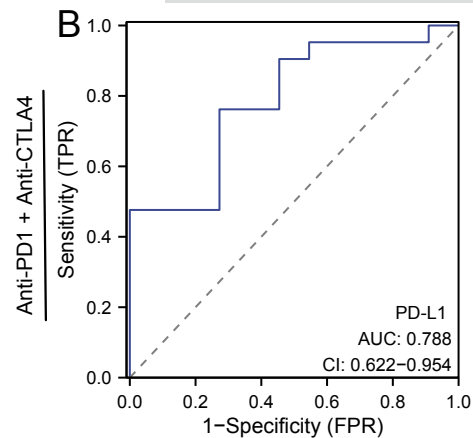**C**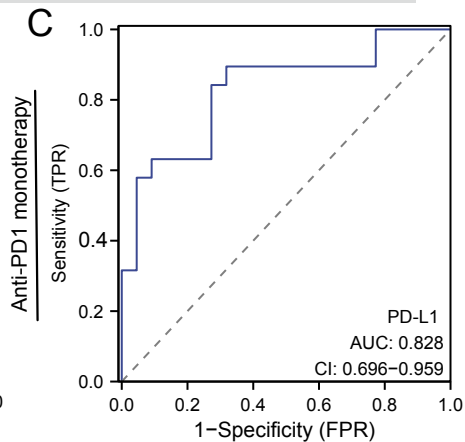**D**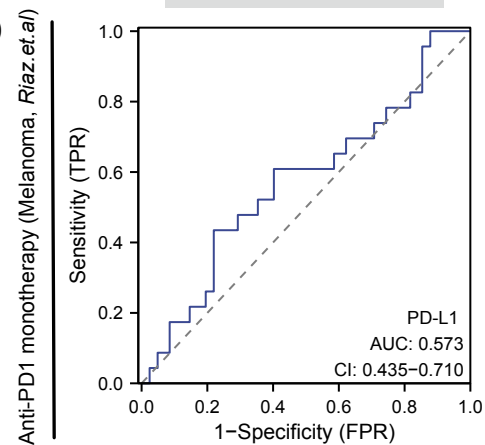

Supplement: Supplementary file 5 — Figure S5 CD103+CD8+ tissue‐resident memory (TRM) predicts response to immune checkpoint blockade (ICB) therapy in melanoma. (A) Statistical comparison of CD103+CD8+ TRM in tumours with different responses to immunotherapy. (B) ROC curve assessing the predictive value of PD‐L1 expression in the Gide cohort (PD‐1 therapy combined with CTLA‐4 therapy). (C) ROC curve assessing the predictive value of PD‐L1 expression in the Gide cohort (PD‐1 monotherapy). (D) ROC curve assessing the predictive value of PD‐L1 expression in the Riaz cohort (PD‐1 monotherapy). [file CTM2-15-e70464-s005.pdf]
