# Supplementary material for: CD103+CD8+ tissue‐resident memory T lymphocytes of melanoma boost anti‐tumour immunity and predict immunotherapy outcomes
Source: Clin Transl Med. 2025 Sep 23;15(9):e70464. doi: 10.1002/ctm2.70464 (PMC12456097; doi:10.1002/ctm2.70464)
Supplement: Supplementary file 4 — Figure S4 The spatial distribution of CD103+CD8+ tissue‐resident memory (TRM) cells and tertiary lymphoid structures (TLS) is significantly correlated. (A) Immune infiltration analysis of melanoma samples in the TCGA cohort; (B) Correlation analysis between ITGAE expression levels and scores of different immune subsets in the sequencing data of TCGA melanoma samples; (C, D) Co‐localisation of the spatial distribution of CD103+CD8+ TRM cells and B lymphocytes; (E) Single‐cell sequencing analysis of CXCL13 expression in CD103+CD8+ TRM cells and CD103−CD8+ cells; (F) Flow cytometry detection of CXCL13 in CD103+CD8+ TRM cells and CD103−CD8+ cells; (G) Analysis of ligand‐receptor pairs of the CD103+CD8+ TRM subpopulation and CD103−CD8+ T cells; (H–K) Spatial transcriptomics analysis of relevant immune subsets; (L) The corresponding haematoxylin and eosin (H&E) images of the spatial transcriptomics data; (M) Spatial transcriptomics analysis of CD103−CD8+ T cell distribution. [file CTM2-15-e70464-s002.pdf]

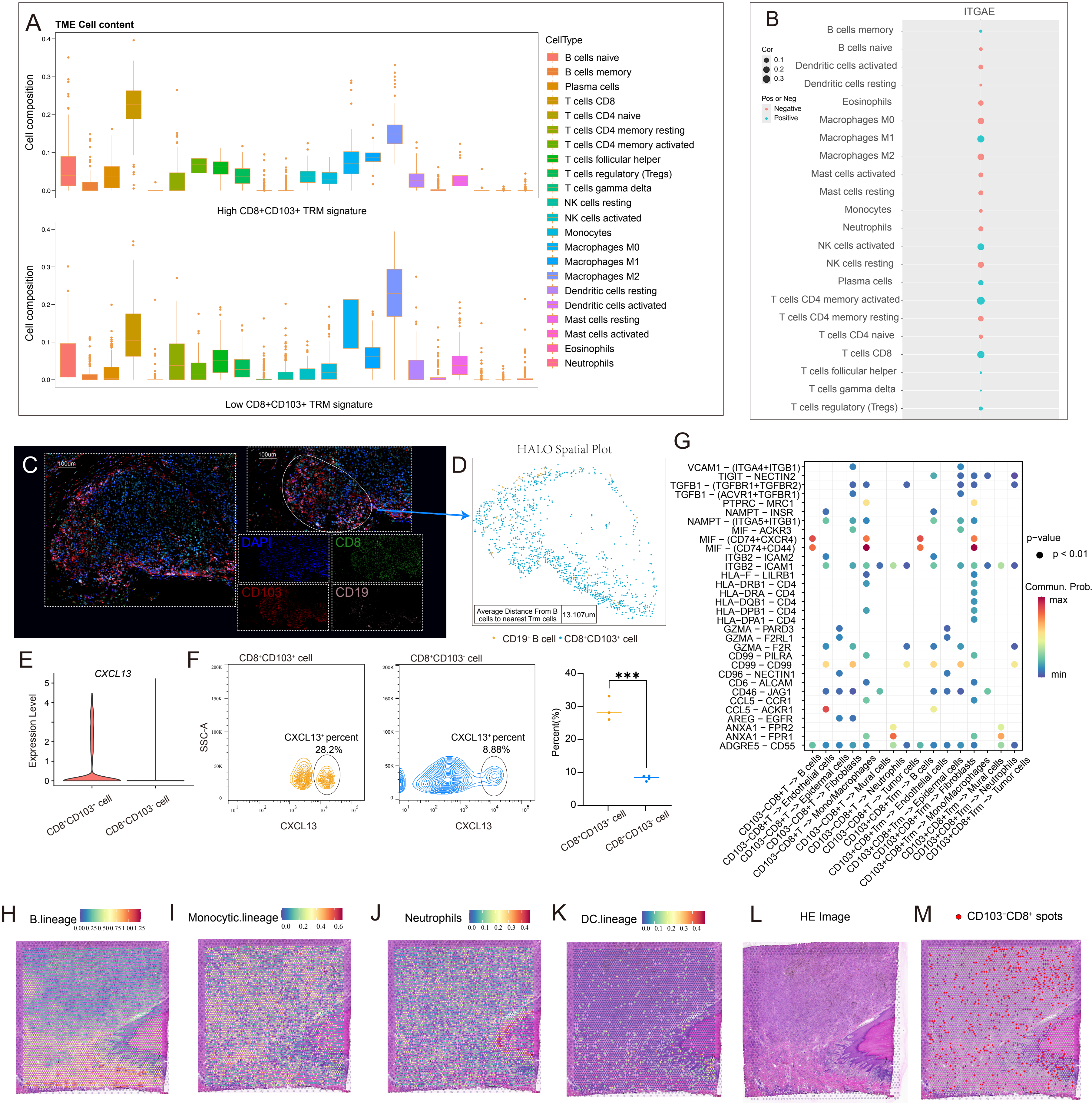

A
TME Cell content
Cell composition
High CD8+CD103+ TRM signature
Low CD8+CD103+ TRM signature
CellType
B cells naive
B cells memory
Plasma cells
T cells CD8
T cells CD4 naive
T cells CD4 memory resting
T cells CD4 memory activated
T cells follicular helper
T cells regulatory (Tregs)
T cells gamma delta
NK cells resting
NK cells activated
Monocytes
Macrophages M0
Macrophages M1
Macrophages M2
Dendritic cells resting
Dendritic cells activated
Mast cells resting
Mast cells activated
Eosinophils
Neutrophils
B
ITGAE
Cor
0.1
0.2
0.3
Pos or Neg
Negative
Positive
C
100um
DAPI
CD8
CD103
CD19
D
HALO Spatial Plot
Average Distance From B cells to nearest Trm cells
13.107um
CD19+ B cell
CD8+CD103+ cell
E
CXCL13
Expression Level
CD8+CD103+ cell
CD8+CD103- cell
F
CD8+CD103+ cell
CD8+CD103- cell
SSC-A
CXCL13
CXCL13+ percent
28.2%
8.88%
Percent(%)
***
G
p-value
p < 0.01
Commun. Prob.
max
min
H
B.lineage
I
Monocytic.lineage
J
Neutrophils
K
DC.lineage
L
HE Image
M
CD103-CD8+ spots
